# Interfacing cellular networks of *S. cerevisiae* and *E. coli*: Connecting dynamic and genetic information

**DOI:** 10.1186/1471-2164-14-324

**Published:** 2013-05-11

**Authors:** Ricardo de Matos Simoes, Matthias Dehmer, Frank Emmert-Streib

**Affiliations:** 1 Computational Biology and Machine Learning Laboratory Center for Cancer Research and Cell Biology School of Medicine, Dentistry and Biomedical Sciences Faculty of Medicine, Health and Life Sciences Queen’s University Belfast 97 Lisburn Road, Belfast, UK; 2 Institute for Bioinformatics and Translational Research UMIT, Hall in Tyrol, Tyrol, Austria

## Abstract

**Background:**

In recent years, various types of cellular networks have penetrated biology and are nowadays used omnipresently for studying eukaryote and prokaryote organisms. Still, the relation and the biological overlap among phenomenological and inferential gene networks, e.g., between the protein interaction network and the gene regulatory network inferred from large-scale transcriptomic data, is largely unexplored.

**Results:**

We provide in this study an in-depth analysis of the structural, functional and chromosomal relationship between a protein-protein network, a transcriptional regulatory network and an inferred gene regulatory network, for *S. cerevisiae* and *E. coli*. Further, we study global and local aspects of these networks and their biological information overlap by comparing, e.g., the functional co-occurrence of Gene Ontology terms by exploiting the available interaction structure among the genes.

**Conclusions:**

Although the individual networks represent different levels of cellular interactions with global structural and functional dissimilarities, we observe crucial functions of their network interfaces for the assembly of protein complexes, proteolysis, transcription, translation, metabolic and regulatory interactions. Overall, our results shed light on the integrability of these networks and their interfacing biological processes.

## Background

With the advent of systems and network biology it is now generally acknowledged that the concerted interactions on all cellular levels between genes and their gene products within a cell are governed by various types of gene networks [1-5]. For instance, the transcriptional regulatory network regulates the expression of genes, whereas protein interaction networks provide a map of diverse types of protein interactions leading, e.g., to the formation of complexes. Unfortunately, large parts of these gene networks are currently unknown leaving us with a fragmented understanding of these networks.

Whereas the first stage of the new era of network biology consisted in the construction and inference of such networks, the next step consists in their analysis, interpretation and integration [6-12]. However, in order to perform an integration of different types of networks, we need to enhance our understanding of the biological information contributed by the individual gene networks as well as their functional overlap. Such an overlap is required because otherwise these networks could not be sensibly integrated with each other due to a lack of common interfacing processes allowing an information flow from one level to the other.

The purpose of the present paper is to study the biological overlap on the genomics and genetics level among three different types of cellular networks, namely the transcriptional regulatory network (TRN), the protein-protein interaction network (PPN) and the gene regulatory network (GRN). A TRN and a PPN are *phenomenological* networks because they are constructed from direct measurements of physical interactions (bindings) between molecular entities, whereas a GRN is an *inferential* network that needs to be statistically inferred from indirect interaction measurements in the form of gene expression data [3]. We study these three cellular networks for *S. cerevisiae* and *E. coli*, because the information available about these organisms is most advanced compared to other, more complex organisms. Besides this, *S. cerevisiae* is the simplest eukaryote system that contains crucial differences in its principle cellular organization compared to prokaryote organisms like *E. coli*. For instance, it is known that the transcription regulation of genes is much more intricate in eukaryotes, which utilize a combinatorial coding of different transcription factors [13,14]. Further, eukaryotes maintain a combination of cis-regulating and trans-acting factors that is absent in prokaryotes [14,15].

Protein-protein networks describe the interactions between proteins in form of physical interactions such as between proteins of a protein complex and transient interactions such as protein modification interactions (e.g. phposphorylation). The majority of experimentally available protein-protein interactions are measured by mass spectrometry methods and large-scale yeast-two-hybrid experiments (Y2H) [16-18]. A number of databases collect protein interaction data from small-scale and large-scale experiments, such as BioGrid [19], IntAct [20], MINT [21], MPact [22] that are also jointly available in meta databases that consider only physical protein interactions [23]. The interactions in a transcriptional regulatory network describe protein-DNA interactions, where transcription factors bind to specific DNA motifs and regulate the gene expression activity of a given target gene. In general, these interactions are measured from protein location data, e.g., from ChIP-Chip or ChIP-seq experiments that are performed for individual transcription factors [24-26].

Finally, gene regulatory networks are inferred networks from large-scale microarray gene expression data sets frequently composed of multiple observational or experimental conditions. The most popular inference methods for gene regulatory networks are based on mutual information [27,28]. A gene regulatory network describes the relationship between gene-pairs based on their mutual dependency of gene expression. Predictions observed in gene regulatory networks have been validated in small-scale experiments for individual transcription factors [29-31]. The gene regulatory network is thus most often equated to the transcriptional regulatory network. However, an inferred gene regulatory network is known to reflect multiple levels of the gene network including also physical interactions, e.g., of proteins belonging to the same protein complex. In our study we distinguish therefore the terminology gene regulatory network from transcriptional regulatory network.

Biological networks that are experimentally derived are given by a binary representation of validated interactions occurring on multiple cellular levels, e.g., between proteins, proteins and DNA, proteins and RNA, RNA and RNA. However, if only one binary network is given, this network lacks the information of the underlying dynamic of temporal and spatial processes that regulate, realize and coordinate the regulatory programs for gene expression, metabolism, growth, differentiation and proliferation of a cell or organism. Hence, it represents the ‘average’ molecular interactions among all these processes. In contrast, the high-throughput data analysis of large-scale gene expression, sequencing or proteomics allows to measure a snapshot from a multitude of specific conditions and cellular states. One major advantage of the inferred interactions of a gene regulatory network is that the cellular context and the average over the underlying condition-specific contexts is considered by large-scale gene expression datasets. The understanding of the relationship of experimental and inferential networks may allow to interpret the role of the cellular and condition-specific contexts from a gene regulatory network in the light of large-scale experimentally evaluated interactions.

In this paper, we investigate phenomenological and inferential cellular networks for *S. cerevisiae* and *E. coli*. More precisely, we compare the structural topology and the functional overlap of the transcriptional regulatory network (TRN), the protein-protein interaction network (PPN) and the gene regulatory network (GRN) for *S. cerevisiae* and *E. coli* on the genomic-scale and the pathway- and interaction-level. Further, we study the genetic connection between interacting genes and the co-localization of genes on the chromosomes. The purpose of our study is to shed light on the integrative abilities of these networks to obtain a multi-level description of the biological processes within a cellular context. We are particularly interested in understanding the role the GRN can play in such an integration. The reason for this is that, so far, discussions about the integration of networks largely exclude the GRN and focus on phenomenological networks like the TRN or the PPN solely. This seems understandable, because a GRN represents an inferred network based on statistical inference algorithms and, in addition, gene expression data used to infer a GRN do not capture any type of *direct* physical interactions among molecules. Instead, they measure merely the concentration of the expression level and, hence, provide *indirect* information about molecular interactions only.

**Figure 1:**
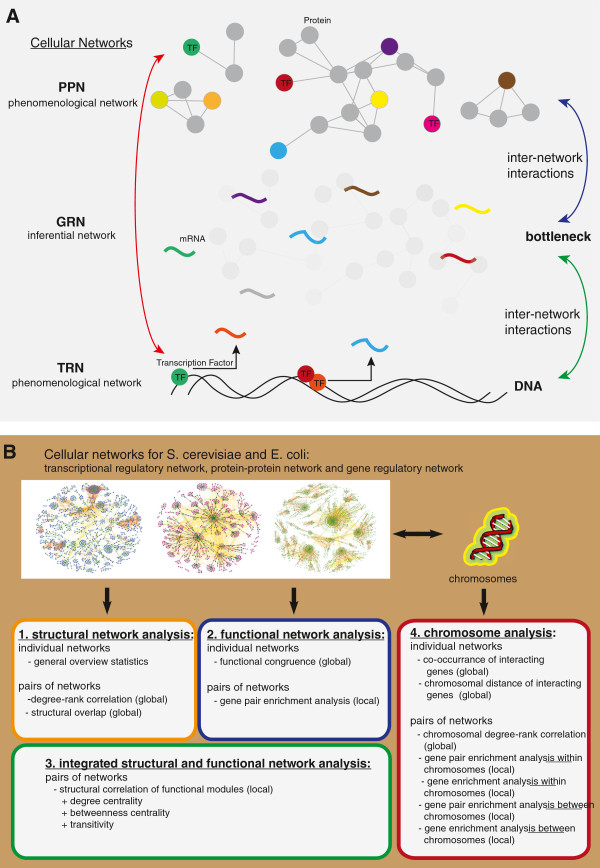
**Overview of the integration of networks and the organization of our analysis. A**: Simplified view of the integration of the transcriptional regulatory network (TRN), gene regulatory network (GRN) and the protein-protein network (PPN) that highlights the pivotal role of the GRN as an interface between the two phenomenological networks. **B**: Principle overview of our analysis. We perform a structural, functional and chromosomal analysis of the TRN, PPN and GRN of *S. cerevisiae* and *E. coli*.

A motivation for our interest in the role of the GRN can be given by a brief description of its principle position within the molecular system (see Figure 1A). In a simplified model, the gene expression is regulated by intrinsic and extrinsic cellular responses regulated by signaling pathways that are defined in a protein network. The downstream response of a signaling pathway can be realized by transcription factors that regulate DNA dependent gene expression, described in the transcription regulatory network. The gene regulatory network can therefore, intuitively, be seen as an *interface* between the protein-protein network and the transcription regulatory network. Hence, the GRN forms a kind of *bottleneck* of the information flow between the TRN and the PPN. The relationship between the three levels of the cellular networks is sketched in Figure 1A, highlighting the interfacing role of the GRN. From this perspective the GRN appears to be important in a sensible integration of phenomenological networks like the TRN and PPN. In this paper, we quantify the relation between the different networks functionally, structurally and genetically.

## Results

Our results section is subdivided into four major parts (see Figure 1B). In part one, we provide a structural analysis of the *S. cerevisiae* and *E. coli* cellular networks. In part two, we conduct a functional network analysis by means of the Gene Ontology (GO) [32] database and in part three, we study structural and functional network features in an integrated manner. Finally, in part four of the results section, we analyze the connection between the chromosomal location of genes and their interconnectedness, as provided by the gene regulatory network (GRN), transcription regulatory network (TRN) and protein-protein interaction network (PPN).

### Structural analysis of the *S. cerevisiae* and *E. coli* cellular networks

#### General overview of the cellular networks

We start, by providing global overview statistics of the GRN, the TRN and the PPN of *S. cerevisiae* and *E.coli*. A summary for *S. cerevisiae* is shown in Table 1A, and for *E. coli* in Table 1B. The GRN of *S. cerevisiae* consists of 9,163 nodes (4,837 genes and 4,326 unmapped probeset ids) and 27,493 edges [33]. The TRN consists of 4,441 genes, of which 157 are transcription factors, and includes a total of 12,873 interactions [34]. The PPN consists of 6,169 genes and 112,562 interactions [23,35]. All three networks have an edge density smaller than 10^−3^. The *E. coli* gene regulatory network consists of 7,258 nodes (4,335 genes, 2,923 transcription units) and 21,820 interactions with a giant connected component (GCC) of 7,064 nodes. The TRN consists of 1,809 genes with 184 transcription factors and includes a total of 3,613 interactions with a GCC of 1,695 genes. Finally, the PPN consists of 3,619 genes and 20,198 interactions with a giant connected component of 3,360 genes. Also the edge densities of these networks is below 10^−3^, as generally observed for gene networks.

**Table 1:** **Summary statistics of the A) S. cerevisiaeand B) *E. coli *gene regulatory network (GRN), protein-protein network (PPN) and transcription regulatory network (TRN)**

**A) S. cerevisiae**					
	**Nodes**	**Edges**	**GCC**	**Edge density**	**Assortativity**
GRN	9,163	27,493	8,978	6.5497e-04	0.0154
PPI	6,169	112,562	6,156	5.9165e-03	-0.5968
TRN	4,441	12,873	4,441	1.3057e-03	-0.1314
**B)*E. coli***					
GRN	7,258	21,820	7,064	8.2853e-04	0.0220
PPI	3,619	20,198	3,360	3.0852e-03	-0.1190
TRN	1,681	3,717	1,585	2.6324e-03	-0.3176

GCC means the giant connected component.

 The network degree assortativity coefficient measures the average degree-degree correlation of connected nodes in a network [36]. A positive coefficient suggests assortative mixing (nodes are likely connected to nodes with similar degree), a negative coefficient disassortative mixing (low degree nodes are likely connected to high degree nodes) and a zero coefficient non-assortative mixing. The assortativity coefficient is close to zero for the GRN (*κ* =0.01) and negative for the PPI (*κ* =−0.5968) and TRN (*κ* =−0.1314)(Table 1).

**Figure 2:**
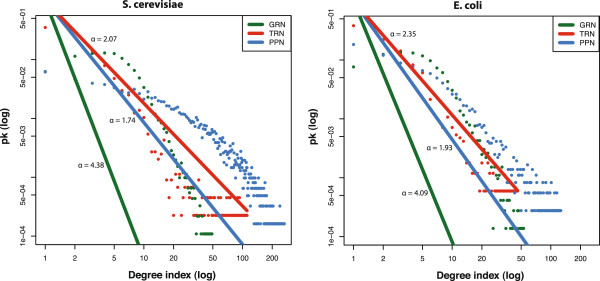
**Comparison of the *log***_**10**_**-*log***_**10**_** degree and fitted power-law distribution for the gene regulatory network (GRN), transcriptional regulatory network (TRN) and protein-protein interaction network (PPI) of *S. cerevisiae *and *E. coli*.**

The degree distribution *p*_*k*_ of a cellular network follows approximately a power law if there is a linear relationship given by, $\log p_{k}=-\alpha \log k+c$, for degree *k*, scaling coefficient *α* and a constant *c*[37]. In Figure 2 we show the degree distribution *p*_*k*_ and the approximated power law for the GRN, TRN and PPN network for *S. cerevisiae* and *E. coli*. The PPN networks show the widest range in the degree values compared to the GRN and TRN. However, the TRN and PPN show stronger similarities in their approximated power law degree distribution (*S. cerevisiaeα*_*trn*_=2.07, *α*_*ppn*_=1.74, *E. coliα*_*trn*_=2.35, *α*_*ppn*_ =1.93) compared to the GRN (*S. cerevisiaeα*_*grn*_=4.38, *E. coliα*_*grn*_=4.09).

#### Pairwise degree-rank, transivity and betweenness correlation

In order to investigate the global structural similarities among the three different types of cellular networks, we perform a pairwise Spearman’s rank correlation test for the degree-ranks, betweenness and transitivity of the genes [38]. We find that between the *S. cerevisiae* gene regulatory network (GRN) and protein-protein interaction network (PPN) the gene graph measures degree rank (*ρ*=0.09, *p* =8.9 *e* −11), betweenness (*ρ* =0.045, *p* =9.4*e* −4) and transitivity (*ρ* =0.025, *p* = 0.04) show a significant correlation. For the pairwise comparisons of the other networks, no significant correlation of the graph measures is observed. For *E. coli* all three pairwise comparisons of the node degrees between the GRN, TRN and PPN do not show any significant rank correlation coefficient, indicating crucial structural differences on a global-level.

#### Structural and functional overlap between the cellular networks

**Figure 3:**
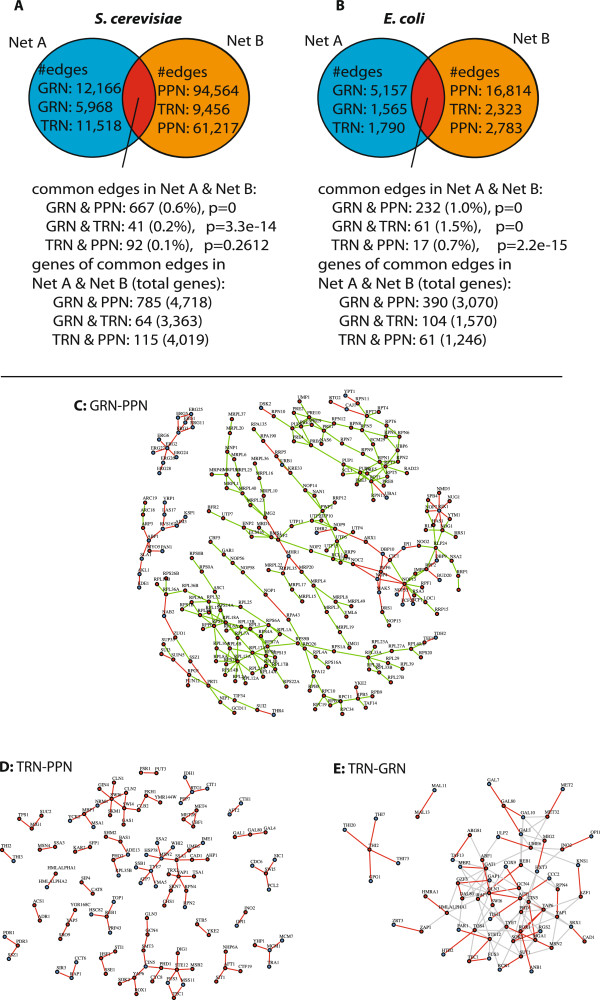
**Structural and functional network analysis of the overlap between pairs of networks.A** and **B**: Pairwise network comparison on the edge-level. **C-E**: Subnetworks of overlapping edges between pairs of cellular networks from *S. cerevisiae*. **C**: (GRN-PPN) Subnetwork of the GRN-PPN interface with 316 of the 667 edges and 248 of the 785 genes. The red nodes correspond to genes annotated to GO:0032991 ‘macromolecular protein complex’ and blue nodes correspond to all other genes. The 242 green edges connect gene pairs that share a term for a protein complex (e.g., ribosome subunit complex) and red edges correspond to all other gene pairs. **D**: (TRN-GRN) Red nodes denote 37 transcription factors and blue nodes 27 target genes. The grey edges are from the TRN and red edges are common between the TRN and GRN. **E**: (TRN-PPN) Green nodes denote 53 transcription factors and the 62 blue nodes are target genes.

The next structural analysis we conduct relates to the structural overlap between the gene regulatory network (GRN), transcriptional regulatory network (TRN) and protein-protein interaction network (PPN) on the interaction-level (edge-level). In the following, we denote the overlap of edges between two cellular networks as their structural interface. The analysis is separately performed for *S. cerevisiae* and *E. coli*. A summary of all network-pair comparisons for *S. cerevisiae* is shown in Figure 3A and for *E. coli* in Figure 3B. For all comparisons, we perform a hypergeometric test to assess whether the overlap between two networks is greater than expected by random chance.

Between the *S. cerevisiae* cellular networks, we observe a percentage of shared edges in the range of 0.1*%* to 0.6*%* (Figure 3A) and for *E. coli* 0.7*%* to 1.5*%* (Figure 3B) between the three networks. Except for the interface of the *S. cerevisiae* TRN and PPN, the number of edges shared between the cellular networks (GRN-PPN, GRN-TRN) are statistically significant. The gene regulatory network (GRN) and the protein-protein network (PPN) show the largest percentage of shared edges of 0.6*%* in *S. cerevisiae*. For *E. coli* the largest percentage of shared edges is 1.5*%* and observed between the gene regulatory network (GRN) and the transcriptional regulatory network (TRN).

For *S. cerevisiae* the GRN and the PPN interface share a total of 667 edges among 785 genes (*p*=0) (Figure 3A and B). The largest connected components (with more than 10 genes) of the shared edges are shown in Figure 3C. The shown subnetworks consist in total of 248 genes and 316 interactions.

The *S. cerevisiae* TRN to GRN interface consists of 0.2*%* shared edges corresponding to 41 edges and 64 genes. The edges correspond to protein-DNA interactions from 37 transcription factors to 27 target genes. (Figure 3D). The genes with the largest degrees are transcription factors with 4 edges *THI2* (thiamine biosynthesis) and 3 edges *AFT1* (iron homeostasis), *CIN5* (stress response), *GAL80* (GAL genes repression), *YAP6* (stress response).

The PPN and the TRN interface share a percentage of 0.1*%* edges that correspond to 92 edges between 115 genes. The edges include 53 transcription factors and 62 target genes (Figure 3E). The genes with the largest degrees are transcription factors with 6 edges *SWI6* (cell cycle) and 5 edges *YAP1* (stress response), *STE12* (MAP kinase signaling), *MSN2* (stress response).

In order to interpret the functional role of shared edges that define an interface between two networks, we performed a Gene Ontology enrichment analysis (GEA) and a gene pair enrichment analysis (GPEA), see ‘Methods’ section, for overlapping edges between two networks for the GO category ‘Biological Process’. The GEA tests for a functional enrichment of a gene set from a collection of connected genes present in a comparison of two cellular networks. In a GEA analysis the genes annotated by the same term are assumed associated to each other. However, the GPEA gives a more objective functional assessment for the individual edges of a network interface rather than the collection of genes participating in the interface. We prefer therefore in this section the detailed assessment of the GPEA analysis over the GEA analysis as the GPEA allows to perform the functional analysis on an edge-centered view compared to the gene-centered view of a GEA analysis. The results of the performed GEA analysis show similar results for all pairwise comparisons (Additional file 1: Figures S1 A,B and Additional file 1: Figure S2).

**Figure 4:**
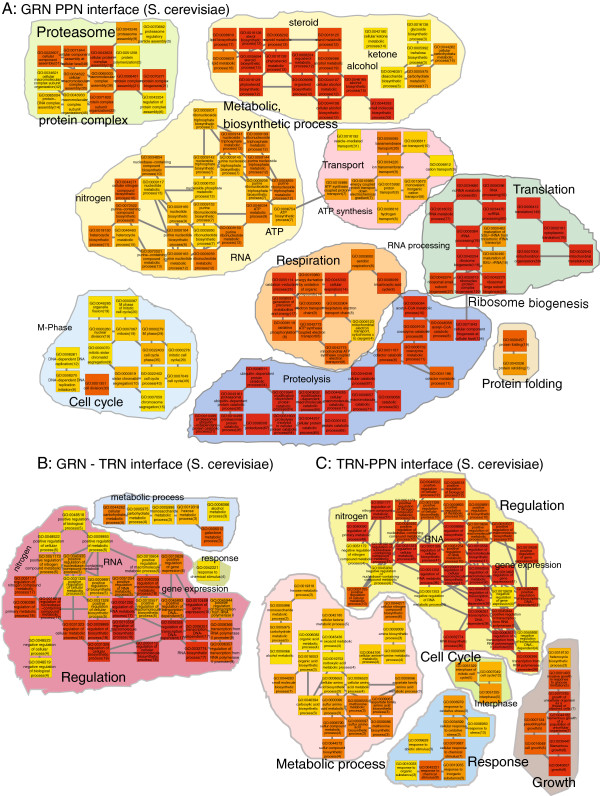
***S. cerevisiae *Biological process GPEA analysis of the network interfaces. A**) GRN-PPN 136 terms (fdr≤1e−4), **B**) GRN-TRN 39 terms (fdr≤0.01), **C**) TRN-PPN 79 terms (fdr≤0.01). The term color gradient denotes high (red) to low rank (yellow), the gene pair number is given in brackets.

**Figure 5:**
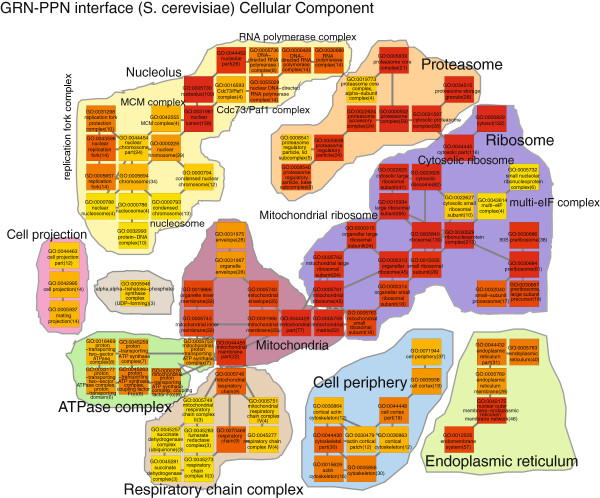
***S. cerevisiae ***GRN-PPN Cellular Component GPEA analysis of the network interfaces. The term color gradient denotes high (red) to low rank (yellow), the gene pair number is given in brackets.

We interpret the results from the GPEA analysis of the network interfaces by constructing a biological process Gene Ontology map for enriched terms of the GRN-PPN interface (Figure 4A), the GRN-TRN interface (Figure 4B) and the TRN-PPN interface (Figure 4C). The *S. cerevisiae* GRN-PPN biological process interface is assessed from 136 terms with *p*_*fdr*_≤1*e*−4 from the total of 302 significant terms *p*_*fdr*_≤0.01. We observe a prominent enrichment for edges involved in the biogenesis of the ribosome, translation of mRNA, proteolysis, proteasome protein complex assembly, metabolic and biosynthetic processes for steroid, alcohol, ketone and lipid, mitochondrial respiration, ATP synthesis and the mitosis M phase of the cell cycle. As we observe a prominent enrichment for protein complex related processes, we also describe in the following the results of the GPEA *S. cerevisiae* GRN-PPN interface analysis using Gene Ontology cellular component terms (Figure 5). We observe a large variety of protein complex GO cellular component terms in the GRN-PPN interface of *S. cerevisiae* such as the MCM complex, the ATP synthase complex, the Cdc73/Paf1 complex, the mitochondrial respiratory chain complex II and IV, the succinate dehydrogenase complex, the fumarate dehydrogenase complex, the RNA polymerase complex, the cytosolic ribosome, the mitochondrial ribosome and the proteasome. The interactions are cytosolic and membrane associated and occur in the mitochodria, nucleus, nuclear outer membrane-endoplasmic reticulum membrane network and cell cortical actin cytoskeloton at the cell periphery.

The GPEA of the S. cerevisiae GRN-TRN interface comprises 39 terms with p_***fdr***_≤0.01 (Figure 4B). The processes involve the positive regulation of metabolic and biosynthetic processes (RNA), regulation of gene expression, galactose and alcohol metabolic process and response to chemical stimulus. The GPEA for the S. cerevisiae TRN-PPN interface shows 79 terms with p_***fdr***_≤0.01 (Figure 4C) and describes biological processes involved in positive regulation of metabolic and biosynthetic processes (e.g., RNA), regulation of gene expression, metabolic processes for alcohol, sulfur, nitrogen, methionine, aspartate, cellular responses to stress, abiotic and organic substances and growth.

The S. cerevisiae GRN-PPN interface shows a close relationship to the actual cellular protein to mRNA interface of cytosolic and mitochondrial ribosomes for protein translation from transcribed mRNA, proteolysis, mitochondrial respiration and cell cycle. In contrast, the network comparisons to the TRN have the biological processes in common that are involved in the regulation of gene expression and biosynthetic and metabolic processes.

Between the E. coli cellular networks, we observe a percentage in the range of 0.7% to 1.5% of shared edges for the pairwise comparisons (Figure 3B). In contrast to the S. cerevisiae networks, we observe for the E.coli networks a higher percentage of shared edges between the GRN and the TRN despite the fact that the absolute number of shared edges is largest between the GRN and the PPN (Figure 3B).

**Figure 6:**
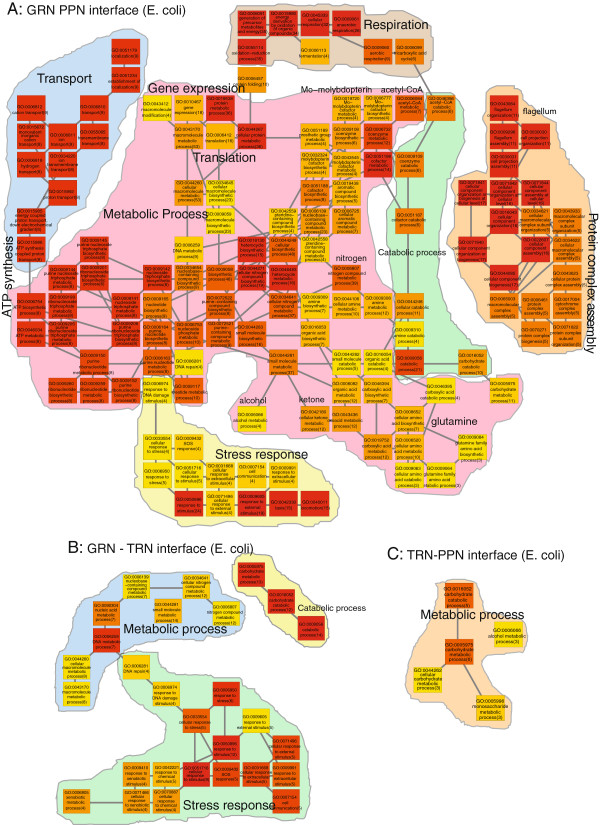
***E. coli ***Biological process GPEA analysis of the network interfaces.A) GRN-PPN 137 terms (fdr≤0.01), B) GRN-TRN 28 terms (fdr≤0.01), C) TRN-PPN 5 terms (fdr ≤ 0.01). The term color gradient denotes high (red) to low rank (yellow), the gene pair number is given in brackets.

For the *E.coli* GRN and the PPN, we observe 232 shared edges (1%) (Figure 3B). The E. coli GRN-PPN GPEA analysis for shared edges shows 137 terms for p_***fdr***_≤0.01. The terms describe biological processes for ion transport for aerobic and anaerobic respiration, ATP synthesis, metabolic processes for glutamine, alcohol, ketone, nitrogen, acetyl-CoA, Mo-molypdopterin, gene expression, translation, protein complex assembly and organization and stress response (Figure 6). Between the GRN and TRN we observe 61 shared edges (1.5%) (Figure 3B). For the E. coli GRN-TRN GPEA interface analysis we observe 28 terms with p_***fdr***_≤0.01 involved in stress response (SOS, DNA damage, chemical stimulus, cell communication), metabolic and catabolic processes. Between the TRN and PPN, we observe the smallest number of 32 shared edges (0.7%) (Figure 3B). We observe for the E. coli TRN-PPN GPEA analysis we observe only 5 terms for carbohydrate and alcohol metabolic processes.

The GEA analysis of the genes in the E. coli interfaces the enrichment shows only terms enriched for the GRN-PPN and only one term for the GRN-TRN genes and is therefore neglected.

The GPEA analysis results compared between S. cerevisiae and E. coli show similarities for the GRN-PPN interface in processes involved in protein complex assembly, respiration, ATP synthesis, translation and metabolic processes. For the GRN-TRN interface between S. cerevisiae and E. coli stress response related processes appear more prominent in E. coli. Compared to the S. cerevisiae GRN-TRN interface in E. coli terms related to response are more apparent rather than terms for describing regulation. The TRN-PPN interface in E. coli shows only 5 terms related to carbohydrate metabolic processes that are also present in the S. cerevisiae TRN-PPN interface.

#### Gene-pair enrichment analysis (GPEA) between cellular networks

In the previous section, we performed a detailed analysis of the functional role of the edges of the interfaces by a GPEA between the GRN, PPN and TRN networks of S. cerevisiae and E. coli. In order to quantify the global functional similarities between the GRN, PPN and TRN, we performed a gene pair enrichment analysis (GPEA) analysis for each of the three networks individually and compare the overlap of significant Gene Ontology terms between them.

Note that in a GPEA interface analysis, we test for the enrichment of edges shared by two networks between the genes in a particular GO term. For a global GPEA analysis that is performed in this section we test for the enrichment of edges in an individual network between the genes in a particular GO term. For each comparison, we include only the subnetwork of genes that are actually present in both networks. For this analysis we used a significance level of α=10^***−4***^ and a Bonferroni adjustment of p-values to correct for multiple testing.

**Figure 7:**
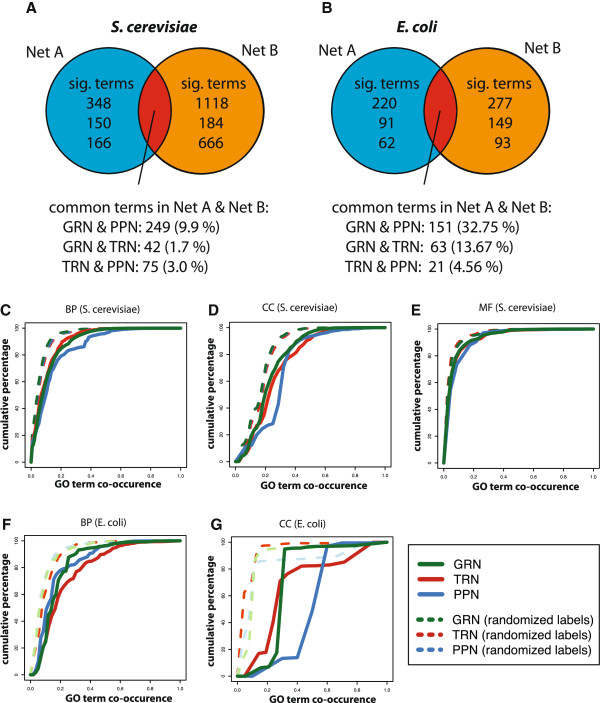
Global functional comparisons between the GRN, TRN and PPN of ***S. cerevisiae ***and ***E. coli***. In A) and B) we show the pairwise comparisons for common significant terms in the global GPEA analysis between the GRN, TRN and PPN for A) S. cerevisiae and B) E. coli. In C) to G) we show the gene-pair distributions sharing a functional annotation for the classes ‘Biological Process’ (BP), ‘Molecular Function’ (MF) and ‘Cellular Component’ (CC). Shown are the cumulative distributions of the co-occurrence frequency of Gene Ontology terms for the edges in the GRN, TRN and PPN in S. cerevisiaeC) BP, D) CC, E) MF and E.coliF) BP, G) CC.

The Figures 7A and B provide a summary of the GPEA analysis of common significant terms between two cellular networks. In both species S. cerevisiae (Sce) and E. coli (Eco) the GRN to PPN comparison shows the largest fractions (Sce 9.9% and Eco 32.75%), GRN to TRN the second largest fractions (Sce 1.7% and Eco 13.67%) and TRN to PPN the smallest fractions (Sce 3.0% and Eco 4.75%) of common significant Gene Ontology terms, significant in both networks. Except for the TRN to PPN comparison the overall fractions are larger in E. coli compared to S. cerevisiae. Although we observe the percentage of shared edges between the E. coli GRN to TRN is larger compared to the E. coli GRN to PPN the GPEA analysis shows a larger functional variety of biological processes in the GRN to PPN comparison as observed for S. cerevisiae.

 In the supplementary, we show a GPEA Gene Ontology map of the common significant terms of the GRN-PPN, GRN-TRN and TRN-PPN comparisons for S.cerevisiae(Additional file 1: Figure S4) and E. coli(Additional file 1: Figure S5). We do not observe major differences between the GPEA interface analysis and the global GPEA analysis for the individual networks. The significant terms describe similar biological processes as described in the previous section for the GPEA analysis of the network interface of a pair of cellular networks.

#### Functional congruence of connected genes within the cellular networks

In order to assess the functional congruence of interactions in the three cellular networks, we quantify the co-occurrence of functional annotations from Gene Ontology for connected genes. More specifically, for each network, we count how often directly connected genes share a common Gene Ontology term. For the comparison, the count frequencies of the co-occurring GO terms are normalized to values between 0 and 1.

In order to compare the results with reference networks, we randomize the gene labels. That means, for each network, we estimate randomized count frequencies from randomizations of the gene labels. In the following, we show the resulting cumulative distributions of the co-occurrence frequency for the three GO categories ‘Biological process’, ‘Cellular component’ and ‘Molecular function’ separately (see Figure 7C-G). For E.coli we excluded the GO category ‘Molecular function’ from the analysis because this category contained only 100 terms.

First, from Figure 7C-G one can see that the three corresponding networks with randomized gene labels (dashed lines) can be distinguished in all cases from the networks with non-randomized gene labels. This indicates that all three cellular networks (GRN, TRN and PPN) contain a considerable amount of biological information about S. cerevisiae and E. coli; because otherwise they would overlap with the results of the networks with randomized gene labels. Due to the fact that, the more GO terms are shared between connected genes, the further the cumulative distributions are shifted to the right (convergence to ‘1.0’ is prolonged), one can see that all three cellular networks contain more information about the GO category ‘Cellular Component’ than ‘Biological Process’ and ‘Molecular Function’.

### Integrated structural and functional analysis of the cellular networks

Next, we perform a local structural comparison on the pathway-level of the cellular networks for S. cerevisiae and E. coli. That means, we conduct an integrated functional and structural analysis of these networks by identifying sets of genes that belong to particular GO terms, for which we assess their structural similarity, by using five graph-based centrality measures and Spearman’s rank correlation test.

As structural network measures, we use (1) degree centrality, (2) betweenness centrality, (3) the local clustering coefficient also called transitivity [39], (4) hubscore centrality and (5) closeness centrality. For our analysis, we consider only GO terms with, 10< genes ≤1000, that are present in two networks. We control the false discovery rate (FDR) at a level of FDR=0.05. The similarity on the gene-set level is measured in the following way. First, we obtain a set of genes that belong to a given GO term, say of p genes. Then, we calculate for these genes one of the five graph-based measures. This results in two p-dimensional vectors whereas the i-th component gives the value of the graph-based measure for the i-th gene. Finally, we compare the similarity of these two vectors by Spearman’s rank correlation test. For all network comparisons the results in Table 2A-C demonstrate that all networks are quite dissimilar on the gene-set level. However, for the S. cerevisiae GRN to PPN comparison we observe a similarity in 32 significant terms for Biological Process using the degree centrality (Table 2A) and 7 terms for Cellular Component using transitivity centrality (Table 2A, D). Although, the number of significant terms is very low for the GRN and PPN network comparison we observe a variety of processes that are related to DNA repair, chromatin remodeling, stress response (e.g., pheromone, arsen), nuclear import/ export, biosynthetic processes (ergosterol, glycogen, ATP) and proteasome (Additional file 1: Figure S5). The 7 significant terms for cellular components between the GRN and PPN of S. cerevisiae comprise terms for proteasome complex, ribosome complex, microtuble associated complex, ligase complex, chromatin and nucleoplasm (Table 2D). The terms partly resemble processes that are observed for the analysis performed on the edge level functional enrichment analysis between the S. cerevisiae GRN and PPN. For the structural comparison of the GRN and PPN the nuclear processes are more pronounced such as stress response, nuclear export/import and chromatin remodeling.

**Table 2:** **Results for *S. cerevisiae***

**A) ***S. cerevisiae*** Biological Process**					
	**Degree**	**Betweenness**	**Transitivity**	**Hubscore**	**Closeness**
GRN/PPN	32/1265	0/1265	2/1265	0/1265	0/1265
TRN/PPN	0/1057	0/1057	0/1057	0/1057	0/1057
GRN/TRN	1/1044	0/1044	1/1044	0/1044	0/1044
**B)*S. cerevisiae* Cellular Component**					
GRN/PPN	4/295	0/295	7/295	5/295	0/295
TRN/PPN	0/245	0/245	0/245	0/245	0/245
GRN/TRN	0/241	0/241	0/241	0/241	0/241
**C)*S. cerevisiae* Molecular Function**					
GRN/PPN	0/375	0/375	5/375	3/375	0/375
TRN/PPN	2/308	0/308	0/308	0/308	0/308
GRN/TRN	0/304	0/304	0/304	0/304	0/304
**D)*S. cerevisiae* transitivity centrality GRN/PPN (Cellular Component)**					
**GO**	**Term**	**Size**	***ρ***	***p***_***fdr***_	
GO:0031597	Cytosolic proteasome complex	202	0.30	0.002024	
GO:0005875	Microtubule associated complex	560	0.16	0.009816	
GO:0019005	SCF ubiquitin ligase complex	26	0.65	0.01706	
GO:0031903	Microbody membrane	17	0.74	0.02134	
GO:0000785	Chromatin	522	0.15	0.02134	
GO:0000313	Organellar ribosome	17	0.74	0.02134	
GO:0005654	Nucleoplasm	12	0.81	0.03283	

Spearman’s rank correlation coefficient test for centrality measures between the GRN, TRN and PPN of Gene Ontology subnetworks for A Biological Process, B Cellular Component and C Molecular Function. A-C Tests are performed for degree, betweenness, transitivity (local clustering coefficient), hubscore and closeness centrality. Each column, shows the number of significant terms and the total number of terms tested. D shows 7 terms for cellular components with significant transitivity centrality between the*S. cerevisiae***GRN**and **PPN**.

 For*E. coli*the results are similar to*S. cerevisiae*, where almost none of the tested pathways showed a significant correlation between pairs of the three networks (GRN, TRN and PPN) for any of the five centrality measures (results not shown).

### Chromosomal co-location and distance of interacting genes

The genes of bacterial species like*E.coli* are organized in an operon structure that have a linear circular genome. In contrast, eukaryotic species like*S. cerevisiae* have a nucleus, where the DNA of the genome is located in form of distinct chromosomes. Hence, eukaryote species have a more complex genomic organization and structure of the genome. Due to the higher complexity of eukaryotic species the evaluation and interpretation of predicted interactions resulting from co-regulation, co-localization or co-expression may be more difficult to judge compared to networks from E.coli. This may be a reason why the relationship of co-localization w.r.t. to the interactions described in cellular networks have not been investigated in great detail so far.

For this reason, we study the chromosomal co-location and the distance between interacting genes for the three networks (GRN, TRN and PPN). First, we estimate the percentages of interactions for genes that are co-located on the same chromosomes and for interactions that correspond to two genes that are located on different chromosomes. For the gene regulatory network, we observe a fraction of 17.14*%* of the interacting genes co-located on the same chromosome, while for the protein-protein network and the transcriptional regulatory network we observe only 8.05*%*** (PPN)** and 7.92*%*** (TRN) **of the interacting genes co-located on the same chromosome.

Next, we study the global degree ranks of the chromosomes for the three networks. The degree ranks of the chromosomes are calculated by the number of interactions corresponding to a particular chromosome. For each chromosome, we count the number of interactions by summing the degrees of each gene corresponding to a particular chromosome. For each network, the chromosomes are then ranked based on the count frequencies. We perform a pairwise comparison using Spearman’s rank correlation test between TRN, GRN and PPN, where we consider only genes present in both networks. From this analysis, we find a significant correlation for all pairwise comparisons (*p*≤2.2***e***−16 with***r*** between 0.84 and 0.88).

Next, we study the co-localization distance of connected gene pairs within chromosomes for the three cellular networks. More precisely, we extract the genomic start and end coordinates of gene-pairs from the chromosomes and calculate their relative distances,* δ *, between connected genes in the GRN, TRN; and PPN. In order to obtain comparable values for chromosomes of differing length,*δ*is normalized by the size of the chromosomes.

**Figure 8:**
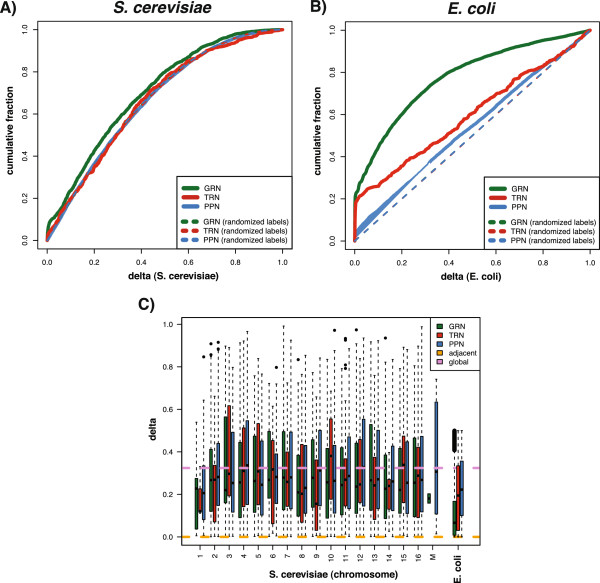
**Cumulative distance distribution of gene pairs for the gene regulatory network (green), transcription regulatory network (red) and protein-protein network (blue). A:***S. cerevisiae*. **B**:*E. coli*. **C**:*S. cerevisiae* relative co-localization distance distribution for each chromosome of gene pairs for the gene regulatory network (GRN), transcription regulatory network (TRN) and protein-protein network (PPN).

In Figure 8, we show the cumulative distance distributions for the GRN, TRN and PPN for*S. cerevisiae* (Figure 8A) and*E. coli* (Figure 8B). To these figures, we added also results from networks with randomized gene labels to contrast the obtained findings. From these figures one can see that the networks of*S. cerevisiae* and*E. coli* behave differently. For*S. cerevisiae*, the TRN and PPN are close to the networks with randomized gene labels, whereas for*E. coli* the difference for the TRN is much larger. That means, e.g., interacting proteins do not have a strong tendency of being co-localized on the same chromosome, similarly, transcription regulation. In contrast, transcription regulation in*E. coli* shows a tendency that the transcription factors and the regulated genes are closer to each other because the cumulative distance distribution for the transcription regulatory network is clearly discernible from the network with randomized gene labels. Statistically, this observation is quantifiable by a two-sample Kolmogorov-Smirnov test.

 Interestingly, the GRN of*S. cerevisiae* and*E. coli*shows the strongest co-localization of connected genes (Figure 8A and B, GRN in green). The reason for this may come from the different nature of this network type, because in contrast to the two phenomenological networks TRN and PPN, the GRN is inferred from gene expression data. In [33,40] it has been shown that such a GRN contains signatures of both phenomenological networks, that means, in the GRN one can find transcription regulations as well as protein-protein interactions. Further, in [41,42] it has been found that inference algorithms used to estimate a GRN favor systematically molecular interactions involving genes having only a moderate number of interactions. In turn, this could hint that genes co-localized on the same chromosome are less connected. 

Figure 8C shows the homogeneity of the relative co-location distance distributions among the chromosomes of*S. cerevisiae* for the GRN, TRN and PPN. In this figure, ‘M’ indicates the mitochondrial chromosome and ‘adjacent’ and ‘global’ provide the average distances over all adjacent gene-pairs on a chromosome respectively all possible gene-pairs regardless of their location on the chromosome. Overall, the average distance between two interacting genes or proteins is around*δ*=0.3 in*S. cerevisiae* and*E. coli* (global) whereas the distance between adjacent genes is below*δ*=0.001. In general, for*S. cerevisiae* the differences between the GRN, TRN and PPN are mild. Only for chromosome 4 (p-value*p*_*bonf*_=4.44*e*−6) and chromosome 12 (*p*_*bonf*_=9.99*e*−03) we obtain a significant difference from a one-way ANOVA testing the equality of the mean distances of the three cellular networks for each chromosome for a significance level of *α*=0.05. This indicates that none of the three networks carries strongly different information about the chromosomes.

#### GEA and GPEA for chromosomal subnetworks

In this last results section, we want to study the functional enrichment of genes for each individual chromosome and gene-pairs of the*S. cerevisiae* cellular networks that are co-located on the same chromosome.

For the GRN, TRN and PPN we perform a Gene Ontology enrichment analysis (GEA) for the category ‘Biological Process’. The GEA is performed for each individual chromosome, using a hypergeometric test, where all genes of a particular network are defined as background. For the GEA analysis, we choose a significance level of*α*=0.01 for nominal p-values to define a set of significant terms for each chromosome. Further, we perform a GPEA analysis for the GRN, TRN and PPN subnetworks of the genes for each individual chromosome. For the GPEA analysis we apply a Bonferroni multiple hypothesis testing correction with a significance level of*α*=0.05. In addition, we estimate the fraction of overlapping significant terms of the GEA and GPEA.

**Figure 9:**
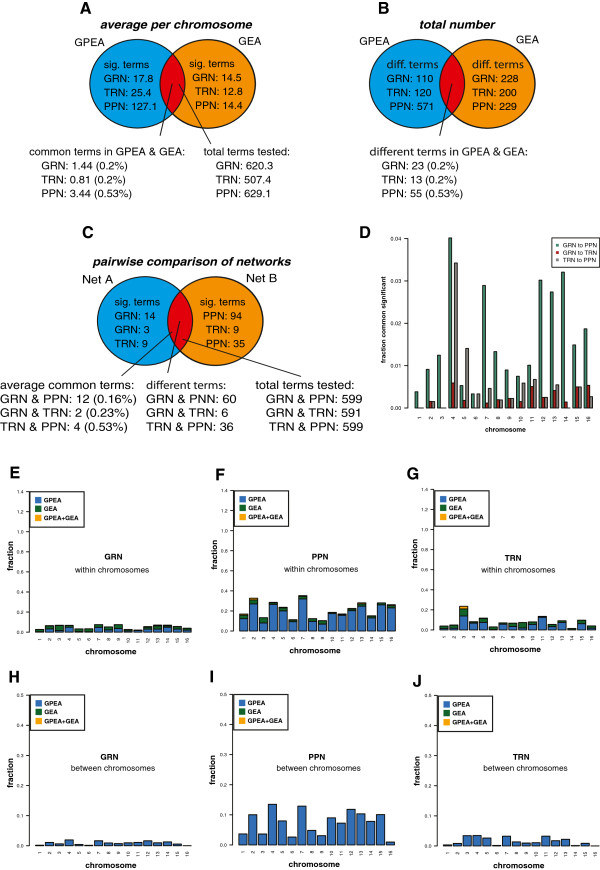
**Results for *S. cerevisiae*.** Summary of the GPEA and GEA, showing average results per chromosome (**A**), total numbers (**B**) of significant terms and pairwise comparison of networks (**C**). **D**: Fraction of common significant terms for the GPEA analysis, between the GRN, TRN and PPN. **E-J**: Fraction of significant GO Biological Process terms for GPEA, GEA and GPEA+GEA overlap analysis for individual chromosomes. **E-G** show results within chromosomes and **H-J** between chromosomes.

In Figure 9A, we show a summary of the chromosome functional enrichment analysis. For the GEA analysis, we observe in average 2% to 5% significant terms. Interestingly, for the GPEA analysis, we observe a large difference in the fraction of significant terms between the GRN and TRN compared to the PPN. For the protein-protein network we observe in average 44% (275 terms) significant GO terms (Biological Process) for each chromosome. For the GRN and TRN the fraction of significant terms are prominently lower showing an average of 2% (17 terms) for the GRN and 5% (24 terms) for the TRN, per chromosome. Further, the number of common different (unique) terms between the GPEA and GEA analysis for the GRN comprises a total of 23 terms, for the TRN we find 10 and for the PPN 143 (see Figure 9A). This indicates that the PPN has a larger function co-localization than the other networks. Here, it is important to emphasize that if one considers interacting proteins in an unselected manner there is no strong co-localization, see Figure 8. However, when restricted to sensibly selected biological subgroups as identified with the Gene Ontology database, there is a strong effect.

In Figure 9E-J, we show an overview of the fractions of significant terms of the GPEA, GEA and their overlap for each chromosome. The fractions for the GRN (Figure 9E), PPN (Figure 9F) and TRN (Figure 9G) show slight variations among the chromosomes.

Finally, we compare the overlap of the functional GPEA analysis between pairs of networks for*S. cerevisiae*. Averaged over all chromosomes, we observe 12 common significant terms between GRN and PPN (1.6*%*), 6 between TRN and PPN (0.85*%*) and 2 between GRN and TRN (Figure 9C). Figure 9D shows more refined results for each chromosomes. The GPEA analysis showing the fractions of the GO terms in the category ‘Biological Process’ that are significant in all pairwise comparisons. The GRN shows a higher similarity with the PPN, compared to the TRN. The topmost common significant terms between the GRN, TRN and PPN are shown in Table 3.

**Table 3:** ***S. cerevisiae *GPEA analysis Biological Process for individual chromosomes**

***S. cerevisiae* GRN to PPN**					
**GOID**	**Term**	**Size**	**Edges1**	**Edges2**	**pval**
GO:0009987	Cellular process	629	389	1509	1.5e-123
GO:0008152	Metabolic process	497	252	1067	7.5e-35
GO:0044237	Cellular metabolic process	481	245	1038	2.25e-33
GO:0044238	Primary metabolic process	450	227	998	1.1e-28
GO:0043170	Macromolecule metabolic process	356	176	854	8.5e-20
GO:0044260	Cellular macromolecule metabolic process	349	173	842	1.15e-19
GO:0006364	rRNA processing	32	18	39	1.2e-13
GO:0016072	rRNA metabolic process	33	18	41	3.45e-13
GO:0042254	Ribosome biogenesis	44	21	56	5.5e-12
GO:0022613	Ribonucleoprotein complex biogenesis	53	23	64	1.05e-10
***S. cerevisiae* GRN to TRN**					
GO:0009987	Cellular process	620	200	241	2.25e-51
GO:0008152	Metabolic process	492	131	147	2.905e-10
GO:0044237	Cellular metabolic process	477	127	143	2.747e-09
GO:0044238	Primary metabolic process	446	122	134	1.235e-08
GO:0043170	Macromolecule metabolic process	356	92	104	0.00291
GO:0044260	Cellular macromolecule metabolic process	349	89	102	0.007955
***S. cerevisiae* TRN to PPN**					
GO:0009987	Cellular process	631	255	1220	6.5e-63
GO:0008152	Metabolic process	499	160	923	4.15e-12
GO:0044237	Cellular metabolic process	483	153	895	1.95e-10
GO:0044238	Primary metabolic process	452	143	855	8e-09
GO:0044249	Cellular biosynthetic process	252	84	351	4.3e-06
GO:0080090	Regulation of primary metabolic process	144	46	222	6.5e-06
GO:0009058	Biosynthetic process	257	85	355	7e-06
GO:0031323	Regulation of cellular metabolic process	142	45	216	9e-06
GO:0010556	Regulation of macromolecule biosynthetic process	117	36	167	1.35e-05
GO:2000112	Regulation of cellular macromolecule biosynthetic process	117	36	167	1.35e-05

Shown are the top 10 common significant terms between the gene regulatory network (GRN), the transcription regulatory network (TRN) and the protein-protein network (PPN). For ranking of terms the corresponding p-values of common significant terms between two networks were averaged. For GRN to TRN comparison only 6 terms were commonly significant.

## Discussion

In this paper, we investigated relations between a transcription regulatory network, a protein-protein network and a gene regulatory network for*S. cerevisiae* and*E. coli*. For these cellular networks, we studied structural, functional and chromosomal properties (I) on the genomic-scale (global) involving the entire network, (II) on the pathway level (local) considering only well defined Gene Ontology terms and (III) on the level of individual interactions. That means our investigation comprised various relevant biological scales of these cellular networks.

From a structural analysis, we found that the three cellular networks (GRN, TRN and PPN) are considerably different from each other. This result is consistent on the genomic-, pathway- and interaction-level. For instance, on the interaction-level, a pairwise comparison between the three networks revealed that the percentage of common interactions (edges) is in general only in the range of 0.1*%* to 1.5*%* percent. This holds for*E. coli* and*S. cerevisiae* (see Figure 3A and B). However, we would like to point out that the GRN and the PPN are more similar to each other than the other two pairs of network combinations, at least for*S. cerevisiae*. An indicator for this has been found, e.g., by the significance of Spearman’s rank correlation coefficient of the degree, betweenness and transitivity distributions.

We studied the functional relationship between the cellular networks by a gene pair enrichment analysis (GPEA) in Gene Ontology terms for the network interfaces that are defined by the set of shared edges between two networks. The functional analysis showed a vast diversity of the biological processes in the network interfaces, although, the fractions of shared edges between the cellular networks are low. The*S. cerevisiae* GRN and PPN interface showed the largest variety of biological processes and protein complexes related to the translation of mRNA (cytosolic and mitochondrial ribosome complex), proteolysis (proteasome complex), metabolic processes, mitochondrial (respiration chain complex II and IV, ATPase synthase complex, succinate dehydrogenase complex), cell cycle (M phase, MCM complex) and transcription (RNA polymerase complex, Cdc73/Paf1 complex). The most prominent and largest Gene Ontology ‘biological processes’ are related to ribosome biogenesis, translation and proteolysis. Proteins are directly synthesized from mRNA and, thus, the identified translational processes corresponds to the*physical interface* between the PPN and the GRN inferred at the mRNA level. Note, that also the proteolysis process is related to the protein translation process, e.g., by post-translational protein processing. Further, the large variety of identified protein complexes in the GRN/PPN interface can be explained by the vital spatial and temporal dependency of genes that belong to the same protein complex to be functional. Protein complexes have been observed to have highly dependent expression profiles [43] and are thus likely to be identified in a gene regulatory network.

For*E. coli* the GPEA analysis of the GRN and PPN interface showed similar results, where we observe biological processes related to protein translation, protein complex assembly and organization, gene expression, aerobic and anaerobic respiration, ATP synthesis, metabolic processes, ion transport and stress response. The majority of the observed biological processes for*E. coli* such as translation, protein complex assembly, metabolic processes, respiration and ATP synthesis are in agreement with the observation for*S. cerevisiae* that indicate to some extend a functional conservation of the GRN-PPN interface between both species.

In*S. cerevisiae* and*E. coli* TRN comparisons, we observe regulatory terms for metabolic processes, gene expression and response that are expected for transcription factor related interactions. For the comparison of the*S. cerevisiae* TRN-GRN the relative low percentages of shared edges are mainly explained by the complex relationships of regulatory protein-DNA interactions that regulate the expression of genes. The observed higher percentage of shared edges in the TRN-GRN compared to the TRN-PPN in both species is reasoned by the closer relationship of the gene expression dependencies inferred by a GRN to transcription factor to target gene interactions. In contrast to*S. cerevisiae*, for*E. coli* the percentage of shared edges of the GRN-TRN is slightly larger than for the GRN-PPN. This may result from the less complex regulation of gene expression in*E. coli*.

The experimental evaluation of single interactions in large inferred networks is very labor and cost intensive. For this reason, a functional co-occurrence of Gene Ontology [32] or pathway annotation is widely used to measure or weight the reliability of interactions between genes [33,40,44]. The principle idea of this approach is based on the concept of*guilt by association* that emerged from the observation that genes with similar expression profiles also tend to share similar biological functions [45]. From a functional co-occurrence analysis of the three networks using Gene Ontology terms from the categories ‘Biological Process’, ‘Cellular Component’ and ‘Molecular Function’, we found that each of the networks contains a considerable amount of biological information, because the biological information content of networks with randomized gene labels can be clearly distinguished (see Figure 7A-E). This is particularly interesting for the GRN, because it demonstrates that the information that can be extracted from such networks is, in terms of its biological knowledge, as valuable as the information extracted from the phenomenological networks (TRN and PPN).

Interestingly, the main difference of the gene regulatory network compared to the phenomenological networks is that a GRN is*inferred* from large-scale data by statistical methods and, thus, a different type of network compared to the TRN and the PPN, which are obtained from direct measurements of molecular interactions, e.g., via ChIP-chip or Y2H experiments. A potential reason for the rich biological information content of the GRN could be given by the way the underlying data are measured, namely*in vivo*. That means, expression data come usually either from cell cultures, tissues or biopsies. In contrast, many PPN are based on yeast two-hybrid measurements that are measured outside a cellular and condition specific context [46].

Finally, we studied genetic information of interacting genes in the three networks. We found that there is a significant difference between the chromosomal co-location and the distance among interacting genes for*E. coli* and*S. cerevisiae*. While for*E. coli* there is a strong co-location effect for close neighbor genes, especially for the TRN and the GRN, this connection is largely absent in*S. cerevisiae* (see Figure 8A and B). This means that in the TRN of*E. coli* transcription factors and the regulated genes are frequently closely located, whereas for*S. cerevisiae* this is not the case. For*S. cerevisiae* this effect is quite homogeneous across all chromosomes (see Figure 8C). Interestingly, the GRN contains the largest fraction of co-localized interacting genes for both organisms, which is around 17%.

From a GPEA and GEA for*S. cerevisiae*, we found that the PPN contains much more chromosome specific interactions than the GRN and the TRN (see Figure 9F and I). This holds for an analysis of interactions within chromosomes (see Figure 9E-G) and between them (see Figure 9H-J). Also, the number of common significant terms is between the GRN and PPN largest compared to all other network pairs (see Figure 9C). This is again an indicator that the GRN contains a considerable amount of information from protein interactions.

## Conclusions

As a summarizing conclusion from the results of our analysis, we hypothesize that the GRN plays a pivotal role when integrating the phenomenological TRN and PPN. The reason for this is that, as seen from our results the overlap between the PPN and TRN is in general much smaller than the overlap between the PPN and the GRN. This holds for a structural, functional and chromosomal analysis, independently, for*E. coli* and*S. cerevisiae*. The reason for this increased overlap comes largely from genes corresponding to the same protein complex [43] and the capability of the BC3NET inference method, used to infer the GRNs in this study, to infer such interactions [33,40]. Hence, a GRN does not only seem to be beneficial as an interface to integrate a TRN with a PPN, but necessary.

Aside from our analysis, this is also plausible for biological reasons, as can be seen in Figure 1, because the data used for the inference of a GRN come from the concentration of mRNAs, which are intermediate between the DNA and the protein level. A further reason in favor for the inclusion of a GRN in such an integration is the type of information represented by the GRN. As explained above, in contrast to the phenomenological TRN and PPN, gene expression data represent the dynamics of the cellular system rather than a static information, because the dynamical concentration levels of mRNAs are converted into a snapshot of the underlying molecular interactions actually happening within these samples. This effect is enlarged by the fact that TRN and PPN are usually generated without considering multiple conditions or outside the cellular context. In contrast, if tissues or biopsies are used as samples to measure the gene expression such data are more representative of the dynamical processes within a cell. However, due to the nature of the employed experimental assays (Y2H or ChIP-chip) neither a PPN nor a TRN alone, or in combination, is sufficient to provide a condition specific map of molecular interactions. Instead, these networks correspond to*cell type specific networks* providing information about potential interactions. However, we hypothesize that if one combines these networks with a*condition specific network*, like the GRN, then the resulting integrated network conveys*condition specific information* induced by the GRN. The reason for this condition specific behavior of a GRN, as discussed above, comes from the way these networks are obtained, namely from inferential methods of*in vivo* samples. This suggests that the integration of different cellular networks should always consist of a combination of*condition specific* and*cell type specific* (*condition unspecific*) networks in order to obtain a phenotype specific model. As shown by our analysis, the observed overlap between the inferential GRN and the two phenomenological networks (PPN and TRN) provides ample opportunities for such an integration.

## Methods

### The BC3NET approach for GRN inference

The BC3NET [33] algorithm is a bagging approach for C3NET [47,48]. The BC3NET algorithm is based on 3 major steps: (1) the generation of bootstrap data sets, (2) inferring ensemble of C3NET networks and (3) aggregation of the network ensemble into a weighted network, where a binomial test is performed for the edges with subsequent consideration for multiple hypothesis testing.

Briefly, the C3NET algorithm selects for each gene at most one edge to a gene neighbor which has the strongest mutual dependency as measured by the mutual information. For each inferred edge, a non-parametric significance test for mutual information is performed. The null distribution for the test is generated by a randomization of the gene expression matrix. We use a Bonferroni multiple hypothesis testing correction with a significance level of*α*=0.05.

From a bootstrap ensemble consisting of 100 data sets a gene regulatory network is inferred using C3NET for each of these data sets. For the network inference, we use a B-spline estimator [49]. A B-spline estimator uses a weighted discretization method to estimate mutual information from continuous values. For each bin, weights are estimated for the corresponding gene expression values from overlapping polynomial B-spline functions. Finally, the ensemble of networks is aggregated into a weighted network, where the weights describe the ensemble consensus rate for an edge. We use a binomial test whether or not an edge should be included in the resulting network. We retain edges for a significance level of*α*=0.05 and a Bonferroni multiple hypothesis testing correction.

### S. cerevisiae and E. coli gene expression data

We use the*S. cerevisiae* Affymetrix ygs98 RMA normalized gene expression compendium available from the Many Microbe Microarrays Database M3D [50]. The yeast compendium dataset comprises 9,335 probesets and 904 samples from experimental and observational data from anaerobic and aerobic growth conditions, gene knockout and drug perturbation experiments. We map the yeast Affymetrix probeset IDs to gene symbols using the annotation of the*ygs98.db* Bioconductor package. Multiple probesets for the same gene are summarized by the median expression value. The resulting expression matrix comprises a total of 9,163 features for 4,837 gene symbols and 4,326 probesets that cannot be assigned to a gene symbol.

Further, we use the***Escherichia coli*** gene expression compendium from the Many Microbe Microarrays Database M3D [50]. The*Escherichia coli* compendium (*version 4, build 6*) comprises a total of 7,459 probesets corresponding to 7,258 unique probeset descriptions and 907 samples. We map the*Escherichia coli* probeset IDs to gene symbols or transcription units from the provided probeset descriptions from M3D. The dataset comprises a total of 4,335 mapped gene symbols and 2,923 mapped transcription units. Multiple probesets for the same gene or transcription unit are summarized by the median expression value.

### Cellular networks of S. cerevisiae and E. coli

#### S. cerevisiae

As gene regulatory network (GRN) we use the BC3NET gene regulatory network described in [33]. This network consists of 9,163 genes and 27,493 edges. The transcriptional regulatory network (TRN) [34] consists of 4,441 genes with 157 transcription factors and includes a total of 12,873 interactions. We use a undirected version of the TRN for our analysis. We map ORF identifiers to gene symbols using the Bioconductor*org.Sc.sgd.db* package. We inferred a BC3NET gene regulatory network from the E. coli gene expression compendium [50] using the B-spline estimator. Finally, the transcription regulatory network for E.coli was assembled from protein-DNA interaction from RegulonDB [51]. The network includes transcription factor to target gene and transcription factor to transcription factor interactions.

#### Protein-Protein interaction network (PPN)

For*Saccharomyces cerevisiae* we use interactions mapped to gene symbols from PINA (*version 2012-12-10*) [23,35]. PINA is a meta database of PPI interaction data from BioGrid [19], DIP [52], IntAct [20], MINT [21] and MPact [22]. The network consists of 6,169 genes and 112,562 interactions.

The PPN for*Escherichia coli* was constructed from binary protein interaction from the DIP (*version 2011-10-27*) [52], IntAct (***version 2012-12***) [20], MINT (*version 2012-10-26*) [21] and MPIDB (*version 2009-11-18*) [53] database. From IntAct, MINT and MPIDB the interactors uniprotkb gene symbols were extracted. For the DIP network uniprotkb, refseq or DIP cross-references to gene symbols were available from EBI or extracted from uniprot entries if the gene symbol was missing (http://www.ebi.ac.uk). The interactions from DIP (12,636 interactions), IntAct (16,517), MINT (5,250) and MPIDB (2,215) were merged resulting in an undirected protein-protein interaction network. The resulting network consists of 3,619 genes and 20,198 interactions.

### Network functional co-occurrence analysis

We compare the edge reliability between inferred and phenomenological cellular networks for*E. coli* and*S. cerevisiae*. The edge reliability is quantified based on the extend of co-occurrence of functional Gene Ontology annotation of connected genes for each network. We compute the cumulative distribution of the number of shared Gene Ontology terms for the edges of the cellular networks for the Gene Ontology classes Biological Process, Molecular Function and Cellular Component.

The extend of co-occurrence is quantified by the count frequency $d_{e_{\text{ij}}}$how often the gene pair*e*_*ij*_ of gene*g*_*i*_ and*g*_*j*_ are described in the same gene set*GO*_*k*_ described by*N* Gene Ontology terms.

(1) $$d_{e_{\text{ij}}}=\overset{N}{\underset{k=1}{\sum }}\left\{\begin{array}{ll} 1 & \text{if}\;g_{i}\in GO_{k},g_{j}\in GO_{k}\\ 0 & \text{else} \end{array}\right)$$

where$d_{e_{\text{ij}}}$ gives the count frequency score of co-occurrence. In the next step we estimate the cumulative distribution from*d* of the$d_{e_{\text{ij}}}$ of all gene pairs to compare the GO co-occurrence between networks. The resulting distribution vector is scaled to the origin. In order to judge the extend of functional co-occurrence that is expected by random chance we randomize gene labels for each network and compute*d*^*r*^ from 100 randomizations.

#### Interfaces between cellular networks

We define the interface between two cellular networks as the subgraph that is induced by the edges that are shared between two networks. The percentages of shared edges between two networks is defined by

(2) $$s_{\text{ij}}=\frac{\left|E_{i}\cap E_{j}\right|}{\left|E_{i}\cup E_{j}\right|}\cdot 100$$

where *E*_*i*_ and *E*_*j*_ define the set of edges in network *i* and *j*.

We perform a hypergeometric test whether the number of shared edges between a pair of networks is larger than expected by random chance. The p-value is estimated using

(3) p=∑i=kmP(X=i)=∑i=kmmiN−mn−iNn

where*m* is the total number of observed interactions of the joined network 1 and network 2,*N* is the number of all possible interactions between the genes shared by both networks and*k*is the number of shared edges between network 1 and network 2.

### Network centrality measures

In the following, we describe network centrality measures degree, hub score, closeness and transitivity that we used for the structural analysis of the cellular networks in our study. The degree of a vertex*v*_*i*_ defines the total number of direct neighbors of*v*_*j*_. For an undirected network the degree of*v*_*i*_ is given by [37]

(4) $$C_{1}(v_{i})=\overset{n}{\underset{j=1}{\sum }}A_{\text{ij}}$$

where*A* is the adjacency matrix of the network.

The closeness centrality of a vertex*v*_*i*_ is defined as the inverse of the mean average shortest path length to all other vertices*v*_*j*_ of a network [54],

(5) $$C_{2}(v_{i})=\frac{1}{\overset{N}{\underset{j=1}{\sum }}d(v_{i},v_{j})}$$

where *i* ≠ *v* and the total number of nodes *N* in the network. If no path exists between two nodes, *d* (*v*,*i*) gives the total number of nodes *N*.

The transitivity centrality of a vertex *v*_*i*_ is a local clustering coefficient that measures the proportion of edges of the direct neighbors of *v*_*i*_ in a clique of *k* vertices where *v*_*j*_ and all its direct neighbors are fully connected. The local clustering coefficient is given by [55]

(6) $$C_{3}(v_{i})=\frac{2|\{e_{\text{ij}}\}|}{k(k-1)}$$

where |*e*_*ij*_| is the number of edges from vertex *v*_*i*_ to all direct neighbors *v*_*j*_and $\frac{k(k-1)}{2}$ gives the total number of edges in the clique of *k* vertices.

In an undirected network the hub score of a vertex *v*_*i*_ is the normalized sum of the hub scores of all direct neighbors *v*_*j*_. The hub score centrality of the vertices in a network are estimated by the principal eigenvector *ω*_1_ of the scalar product of the adjacency matrix *A* and its transpose [56].

(7) $$\omega_{1}(A\cdot t(A))$$

### Network pathway analysis using centrality measures

For the comparison between two networks, we consider only the subnetworks of common genes. For two cellular networks,*G*_*a*_ and*G*_*b*_, we estimate the degree, betweenness, transitivity, hubscore and closeness centrality values for all genes for a Gene Ontology (GO) term. Then, for each GO term, we perform a Spearman’s rank correlation test [38] for the ranks of the values for each centrality measure between a pair of networks. We adjust p-values using a FDR [57] correction for a given significance level of*α*=0.05.

### Gene ontology enrichment analysis and annotation

We use Gene Ontology annotation using the Bioconductor [58] package *org.Sc.sgd.db* for *S. cerevisiae* and *org.EcK12.eg.db* for*E. coli*. Gene Ontology terms and class definitions (BP, MF, CC) were extracted from the *GO.db* Bioconductor package. The Gene Ontology enrichment analysis (GEA) was performed with the*topGO* package [59] using a hypergeometric test.

### Gene Pair Enrichment Analysis (GPEA)

We test for the enrichment of gene pairs connected in a network sharing the same Gene Ontology term annotation. For each Gene Ontology term we perform a hypergeometric test (one-sided Fisher exact test) for edges (gene pairs). For *p* genes a total of *N* = *p*(*p* −1)/2 possible gene pairs can be formed. A set of genes annotated by a GO term *p*_*GO*_ form a total of *m* = *p*_*GO*_(*p*_*GO*_−1)/2 possible gene pairs. From a cellular network with *n* edges the subnetwork for each GO term with*k* edges is considered. The p-value for the enrichment of this GO-term is calculated from a hypergeometric distribution by

(8) p=∑i=kmP(X=i)=∑i=kmmiN−mn−iNn

The p-value gives an estimate of the probability to observe *k* or more edges between genes from the given GO-term. For the analysis we consider Gene Ontology Biological Process terms with more than 2 and less than 1000 genes. The p-values are adjusted using a bonferroni multiple hypothesis testing procedure. We select terms significant with *p*_*bonferroni*_ = 0.0001.

In the following we describe the GPEA analysis for functional gene pair enrichment of shared edges between two networks. For each Gene Ontology term we perform a hypergeometric test (one-sided Fisher exact test) for the enrichment of gene pairs sharing the same functional annotation between two networks (analog to eqn. 8). For *p* genes the joint number of edges for two networks is given by$N=G_{1}^{e}\cup G_{2}^{e}$. The total number of *n* edges common between two networks is given by$n=G_{1}\cap G_{2}$. The joint number of *m* edges of two subnetworks *S* for a GO term*t* is given by$m=S_{t}(G_{1}^{e}\cup G_{2}^{e})$. The number of edges of the subnetwork *S* common between two networks for a GO term *t* is given by$k=S_{t}^{e}(G_{1}^{e}\cap G_{2}^{e})$. The p-value gives an estimate of the probability to observe *k* or more edges between genes from the given GO-term. For the analysis we consider Gene Ontology Biological Process terms with more than 2 and less than 500 genes. The p-values are adjusted using a Benjamini Hochberg (fdr) multiple hypothesis testing procedure. We select terms significant with *p*_*fdr*_ = 0.01.

### Gene ontology graph visualization

For the visualization of the Gene Ontology graphs we use our currently unpublished R-Package *drawgo*. For a set of defined Gene Ontology terms a Gene Ontology subgraph is extracted from Gene Ontology including the set of significant Gene Ontology terms and the corresponding parental terms [60]. In order to reduce the size of the GO graph for a visualization of the graph we delete iteratively non-significant parental terms from the graph. The corresponding child terms of a deleted parental GO term are connected to the corresponding parent GO terms of the deleted parental GO term of the graph. In the visualization the connections between Gene Ontology terms do not necessarily show direct parent child connections and also include more distant ancestor child connection when non-significant direct parents were deleted. The layout in drawgo is based on a force-based grid layout for the visualization. The graph procedures and visualization is based onigraph [61].

### Relative gene location distance

We retrieved the *E. coli K12* Genebank refSeq coordinates from the UCSC Microbial Genome Browser [62]. We define the relative distance *δ*∈[0,1] between two genes *g*_*i*_ and *g*_*j*_that are co-located on the same chromosome by the distance between the mid points of the two genes normalized by the size of the chromosome.

The mid point coordinate of a gene is given by

(9) $$m(g_{i})=\text{start}(g_{i})+\frac{\text{end}(g_{i})-\text{start}(g_{i})}{2}$$

where*end*(*g*_*i*_)≥*start*(*g*_*i*_). start() gives the start and end() the physical end coordinate in bp (base pair) units.

The distance between two genes in a circular genome is defined by

(10) $$\delta =\text{min}\left\{\begin{array}{l} \frac{m(g_{j})-m(g_{i})}{L_{k}}\\ \frac{L_{k}-m(g_{j})-m(g_{i})}{L_{k}} \end{array}\right)$$

for*m*(*g*_*j*_)>*m*(*g*_*i*_).*L*_*k*_is the chromosome size of chromosome *k*in bp where *g*_*i*_ and *g*_*j*_ are co-located.

## Competing interests

The authors declare that they have no competing interests.

## Authors’ contributions

FES conceived and designed the study. RDMS and FES performed the analysis. FES, RDMS and MD interpreted the results and wrote the paper. All authors read and approved the final manuscript.

## Supplementary Material

Additional file 1 **Supplementary file.** Interfacing cellular networks of *S. cerevisiae* and *E. coli*: Connecting dynamic and genetic information. Click here for file

## References

[B1] Alon U An Introduction to Systems Biology: Design Principles of Biological Circuits 2006 Boca Raton: Chapman & Hall/CRC

[B2] Barabasi A Oltvai Z Network biology: understanding the cell’s functional organization Nat Rev Genet 2004 5 101 13 10.1038/nrg1272 14735121

[B3] Emmert-Streib F Glazko G Network Biology: A direct approach to study biological function Wiley Interdiscip Rev Syst Biol Med 2011 3 4 379 391 10.1002/wsbm.134 21197659

[B4] Palsson B Systems Biology 2006 Cambridge, New York: Cambridge University, Press

[B5] Vidal M A unifying view of 21st century systems biology FEBS Let 2009 583 24 3891 3894 10.1016/j.febslet.2009.11.024 19913537

[B6] Bebek G Koyuturk M Price ND Chance MR Network biology methods integrating biological data for translational science Brief Bioinform 2012 13 4 446 459 10.1093/bib/bbr075 22390873 PMC3404396

[B7] Emmert-Streib F Analysis of Complex Networks: From Biology to Linguistics 2009 Weinheim: Wiley-VCH

[B8] Emmert-Streib F Dehmer M Networks for Systems biology: conceptual connection of data and function IET Syst Biol 2011 5 3 185 10.1049/iet-syb.2010.0025 21639592

[B9] Hwang D Rust A Ramsey S Smith J Leslie D Weston A de Atauri P Aitchison J Hood L Siegel A Bolouri H A data integration methodology for systems biology Proc Natl Acad Sci USA 2005 102 48 17296 172301 10.1073/pnas.0508647102 16301537 PMC1297682

[B10] Mueller L Kugler K Graber A Emmert-Streib F Dehmer M Structural measures for network biology Using QuACN BMC Bioinformatics 2011 12 1 492 10.1186/1471-2105-12-492 22195644 PMC3293850

[B11] Wang Y Chen B Integrated cellular network of transcription regulations and protein-protein interactions BMC Syst Biol 2010 4 20 10.1186/1752-0509-4-20 20211003 PMC2848195

[B12] Yeger-Lotem E Margalit H Detection of regulatory circuits by integrating the cellular networks of protein-protein interactions and transcription regulation Nucleic Acids, Res 2003 31 6053 6061 10.1093/nar/gkg787 14530453 PMC219468

[B13] Chen L Combinatorial gene regulation by eukaryotic transcription factors Curr Opin Struct Biol 1999 9 1 48 55 10.1016/S0959-440X(99)80007-4 10047576

[B14] Harbison CT Gordon DB Lee TI Rinaldi NJ Macisaac KD Danford TW Hannett NM Reynolds DB Yoo J Tagne J-b Transcriptional regulatory code of a eukaryotic genome Nature 2004 431 7004 99 104 10.1038/nature02800 15343339 PMC3006441

[B15] Barreram LO Ren B The transcriptional regulatory code of eukaryotic cells–insights from genome-wide analysis of chromatin organization and transcription factor binding Curr Opin Struct Biol 2006 18 3 291 298 10.1016/j.ceb.2006.04.002 16647254

[B16] Bantscheff M Schirle M Sweetman G Rick J Kuster B Quantitative mass spectrometry in proteomics: a critical reviewem Anal, Bioanal Chem 2007 389 4 1017 1031 10.1007/s00216-007-1486-6 17668192

[B17] Koegl M Uetz P Improving yeast two-hybrid screening systems Brief Funct, Genomic Proteomic 2007 6 4 302 312 18218650 10.1093/bfgp/elm035

[B18] Vidal M Cusick ME Barabási A-L Interactome networks and human disease Cell 2011 144 6 986 998 10.1016/j.cell.2011.02.016 21414488 PMC3102045

[B19] Stark C Breitkreutz B Reguly T Boucher L Breitkreutz A Tyers M BioGRID: a general repository for interaction datasets Nucleic Acids Res 2006 34 D535 9 10.1093/nar/gkj109 16381927 PMC1347471

[B20] Aranda B Achuthan P Alam-Faruque Y Armean I Bridge A Derow C Feuermann M Ghanbarian A Kerrien S Khadake J Kerssemakers J Leroy C Menden M Michaut M Montecchi-Palazzi L Neuhauser S Orchard S Perreau V Roechert B van Eijk K and Hermjakob H The IntAct molecular interaction database in 2010 Nucleic Acids Res 2010 38 D525 D531 10.1093/nar/gkp878 19850723 PMC2808934

[B21] Licata L Briganti L Peluso D Perfetto L Iannuccelli M Galeota E Palma A Nardozza A Santonico E Castagnoli L Sacco F MINT, the molecular interaction database 2012 update Nucleic Acids Res 2012 40 D857 D861 10.1093/nar/gkr930 22096227 PMC3244991

[B22] Guldener U Munsterkotter M Oesterheld M Pagel P Ruepp A Mewes H Stumpflen V MPact: the MIPS protein interaction resource on yeast Nucleic Acids Res 2006 34 D436 D441 10.1093/nar/gkj003 16381906 PMC1347366

[B23] Wu J Vallenius T Ovaska K Westermarck J Makela T Hautaniemi S Integrated network analysis platform for protein-protein interactions Nat Methods 2009 6 75 77 10.1038/nmeth.1282 19079255

[B24] Buck M Lieb J Chip-chip: considerations for the design, analysis, and application of genome-wide chromatin immunoprecipitation experiments Genomics 2004 83 3 349 360 10.1016/j.ygeno.2003.11.004 14986705

[B25] Kidder BL Hu G Zhao K ChIP-Seq: technical considerations for obtaining high-quality data Nat Immunol 2011 12 10 918 922 10.1038/ni.2117 21934668 PMC3541830

[B26] Park K Kim D Localized network centrality and essentiality in the yeast-protein interaction network Proteomics 2009 9 22 5143 5154 10.1002/pmic.200900357 19771559

[B27] Cover T Thomas J Information Theory 1991 John Wiley & Sons, Inc

[B28] Emmert-Streib F Glazko G Altay G de Matos Simoes R Statistical inference and reverse engineering of gene regulatory networks from observational expression data Front Genet 2012 3 8 22408642 10.3389/fgene.2012.00008 PMC3271232

[B29] Basso K Margolin A Stolovitzky G Klein U Dalla-Favera R Califano A Reverse engineering of regulatory networks in human B cells Nat Genet 2005 37 382 390 10.1038/ng1532 15778709

[B30] Faith J Hayete B Thaden J Mogno I Wierzbowski J Cottarel G Kasif S Collins J Gardner T Large-scale mapping and validation of Escherichia coli transcriptional regulation from a compendium of expression profiles PLoS Biol 2007 5 e8 10.1371/journal.pbio.0050008 17214507 PMC1764438

[B31] Margolin A Nemenman I Basso K Wiggins C Stolovitzky G Dalla Favera R Califano A ARACNE: an algorithm for the reconstruction of gene regulatory networks in a mammalian cellular context BMC Bioinformatics 2006 7 Suppl 1 S7 10.1186/1471-2105-7-S1-S7 16723010 PMC1810318

[B32] Ashburner M Ball C Blake J Botstein D Butler H Cherry J Davis A Dolinski K Dwight S Eppig J Harris M Hill D Issel-Tarver L Kasarskis A Lewis S Matese J Richardson J Ringwald M Rubin G Sherlock G Gene ontology: tool for the unification of biology. The gene ontology consortium Nat Genet 2000 25 25 29 10.1038/75556 10802651 PMC3037419

[B33] de Matos Simoes R Emmert-Streib F Bagging statistical network inference from large-scale gene expression data PLoS ONE 2012 7 3 e33624 10.1371/journal.pone.0033624 22479422 PMC3316596

[B34] Balaji S Babu M Iyer L Luscombe N Aravind L Comprehensive analysis of combinatorial regulation using the transcriptional regulatory network of yeast J Mol Biol 2006 360 213 227 10.1016/j.jmb.2006.04.029 16762362

[B35] Cowley M Pinese M Kassahn K Waddell N Pearson J Grimmond S Biankin A Hautaniemi S Wu J PINA v2.0: mining interactome modules Nucleic Acids Res 2012 40 D862 D865 10.1093/nar/gkr967 22067443 PMC3244997

[B36] Newman M Assortative mixing in networks Phys Rev Lett 2002 89 208701 12443515 10.1103/PhysRevLett.89.208701

[B37] Newman M Networks: An Introduction 2010 Oxford: Oxford University Press

[B38] Sheskin DJ Handbook of Parametric and Nonparametric Statistical Procedures 2004 Boca Raton: RC Press

[B39] Newman MEJ The structure and function of complex networks SIAM Rev 2003 45 167 256 10.1137/S003614450342480

[B40] de Matos Simoes R Tripathi S Emmert-Streib F Organizational structure of the peripheral gene regulatory network in B-cell lymphoma BMC Syst Biol 2012 6 38 10.1186/1752-0509-6-38 22583750 PMC3476434

[B41] Altay G Emmert-Streib F Revealing differences in gene network inference algorithms on the network-level by ensemble methods Bioinformatics 2010 26 14 1738 1744 10.1093/bioinformatics/btq259 20501553

[B42] Emmert-Streib F Altay G Local network-based measures to assess the inferability of different regulatory networks IET Syst Biol 2010 4 4 277 288 10.1049/iet-syb.2010.0028 20632777

[B43] Jansen R Greenbaum D Gerstein M Relating whole-genome expression data with protein-protein interactions Genome Res 2002 12 37 46 10.1101/gr.205602 11779829 PMC155252

[B44] Madhamshettiwar P Maetschke S Davis M Reverter A Ragan M Gene regulatory network inference: evaluation and application to ovarian cancer allows the prioritization of drug targets Gen Med 2012 4 5 41 10.1186/gm340 PMC3506907 22548828

[B45] Wolfe C Kohane I Butte A Systematic survey reveals general applicability of “guilt-by-association” within gene coexpression networks BMC Bioinformatics 2005 6 227 10.1186/1471-2105-6-227 16162296 PMC1239911

[B46] Maslov S Sneppen K Specificity and stability in topology of protein networks Science 2002 296 910 913 10.1126/science.1065103 11988575

[B47] Altay G Emmert-Streib F Inferring the conservative causal core of gene regulatory networks BMC Syst Biol 2010 4 132 10.1186/1752-0509-4-132 20920161 PMC2955605

[B48] Altay G Emmert-Streib F Structural Influence of gene networks on their inference: Analysis of C3NET Biol Direct 2011 6 31 10.1186/1745-6150-6-31 21696592 PMC3136421

[B49] Daub C Steuer R Selbig J Kloska S Estimating mutual information using B-spline functions–an improved similarity measure for analysing gene expression data BMC Bioinformatics 2004 5 118 10.1186/1471-2105-5-118 15339346 PMC516800

[B50] Faith J Driscoll M Fusaro V Cosgrove E Hayete B Juhn F Schneider S Gardner T Many Microbe Microarrays Database: uniformly normalized Affymetrix compendia with structured experimental metadata Nucleic Acids Res 2008 36 D866 D870 17932051 10.1093/nar/gkm815 PMC2238822

[B51] Gama-Castro S Salgado H Peralta-Gil M Santos-Zavaleta A Muniz-Rascado L Solano-Lira H Jimenez-Jacinto V Weiss V Garcia-Sotelo J Lopez-Fuentes A Porron-Sotelo L Alquicira-Hernandez S Medina-Rivera A Martinez-Flores I Alquicira-Hernandez K Martinez-Adame R Bonavides-Martinez C Miranda-Rios J Huerta A Mendoza-Vargas A Collado-Torres L Taboada B Vega-Alvarado L Olvera M Olvera L Grande R Morett E Collado-Vides J RegulonDB version 7.0: transcriptional regulation of Escherichia coli K-12 integrated within genetic sensory response units (Gensor Units) Nucleic Acids Res 2011 39 D98 D105 10.1093/nar/gkq1110 21051347 PMC3013702

[B52] Xenarios I Salwinski L Duan X Higney P Kim S Eisenberg D DIP, the database of interacting proteins: a research tool for studying cellular networks of protein interactions Nucleic Acids Res 2002 30 303 30s5 10.1093/nar/30.1.303 11752321 PMC99070

[B53] Goll J Rajagopala S Shiau S Wu H Lamb B Uetz P MPIDB: the microbial protein interaction database Bioinformatics 2008 24 1743 1744 10.1093/bioinformatics/btn285 18556668 PMC2638870

[B54] Freeman LC Centrality in social networks: Conceptual clarification Social Networks 1979 1 215 239

[B55] Watts D Strogatz S Collective dynamics of ’small-world’ networks Nature 1998 393 440 442 10.1038/30918 9623998

[B56] Kleinberg JM Authoritative sources in a hyperlinked environment J ACM Sept 1999 46 5 604 632 10.1145/324133.324140

[B57] Benjamini Y Hochberg Y Controlling the false discovery rate: a practical and powerful approach to multiple testing J Royal Stat Soc, Ser B (Methodological) 1995 57 125 133

[B58] Gentleman R Carey V Bates D Bolstad B Dettling M Dudoit S Ellis B Gautier L Ge Y Gentry J Hornik K Hothorn T Huber W Iacus S Irizarry R Leisch F Li C Maechler M Rossini A Sawitzki G Smith C Smyth G Tierney L Yang J Zhang J Bioconductor: open software development for computational biology and bioinformatics Genome Biol 2004 5 R80 10.1186/gb-2004-5-10-r80 15461798 PMC545600

[B59] Alexa A Rahnenfuhrer J Lengauer T Improved scoring of functional groups from gene expression data by decorrelating GO graph structure Bioinformatics 2006 22 1600 1607 10.1093/bioinformatics/btl140 16606683

[B60] Beissbarth T Speed T GOstat: find statistically overrepresented Gene Ontologies within a group of genes Bioinformatics 2004 20 1464 1465 10.1093/bioinformatics/bth088 14962934

[B61] Csardi G Nepusz Tigraph-package 2008

[B62] Schneider K Pollard K Baertsch R Pohl A Lowe T The UCSC archaeal genome browser Nucleic Acids Res 2006 34 D407 D410 10.1093/nar/gkj134 16381898 PMC1347496

