# Supplementary material for: Interfacing cellular networks of S. cerevisiae and E. coli: Connecting dynamic and genetic information
Source: BMC Genomics. 2013 May 11;14:324. doi: 10.1186/1471-2164-14-324 (PMC3698017; doi:10.1186/1471-2164-14-324)
Supplement: Additional file 1 — Supplementary file. Interfacing cellular networks of S. cerevisiae and E. coli: Connecting dynamic and genetic information. [file 1471-2164-14-324-S1.pdf]

Supplementary file:
Interfacing cellular networks of *S. cerevisiae* and *E. coli*: Connecting dynamic and genetic information

Ricardo de Matos Simoes[1], Matthias Dehmer[2], Frank Emmert-Streib[1*]
[1]Computational Biology and Machine Learning Laboratory
Center for Cancer Research and Cell Biology
School of Medicine, Dentistry and Biomedical Sciences
Faculty of Medicine, Health and Life Sciences
Queen's University Belfast
97 Lisburn Road, Belfast, UK
[2] Institute for Bioinformatics and Translational Research
UMIT, Hall in Tyrol, Austria

*corresponding author: v@bio-complexity.com

A) GEA GRN-PPN interface (S. cerevisiae)

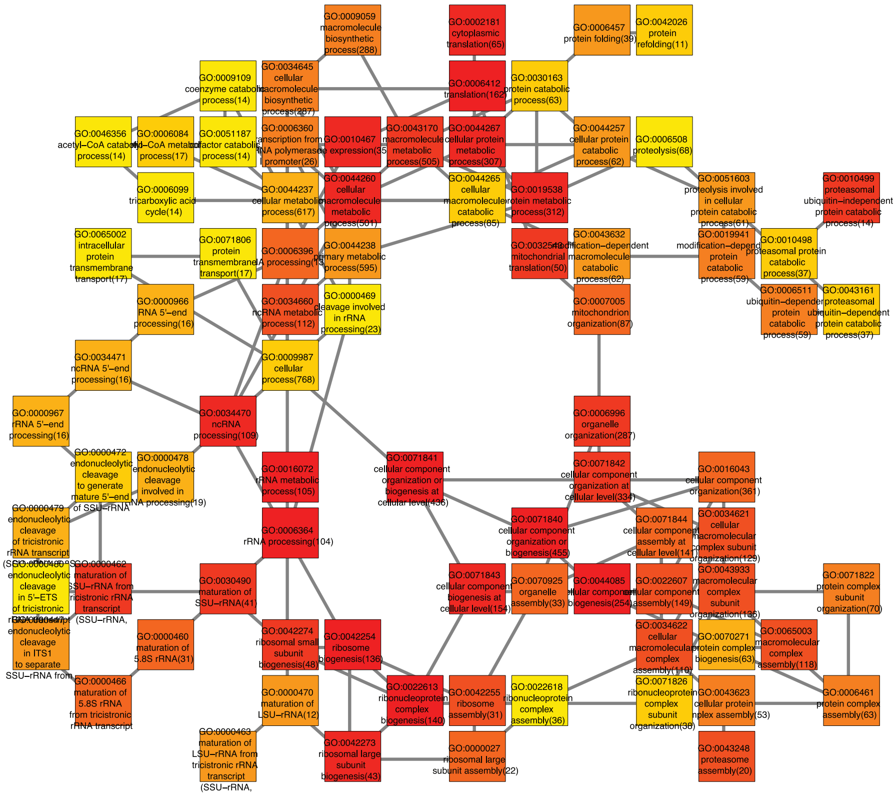

B) GEA GRN-TRN interface (S. cerevisiae)

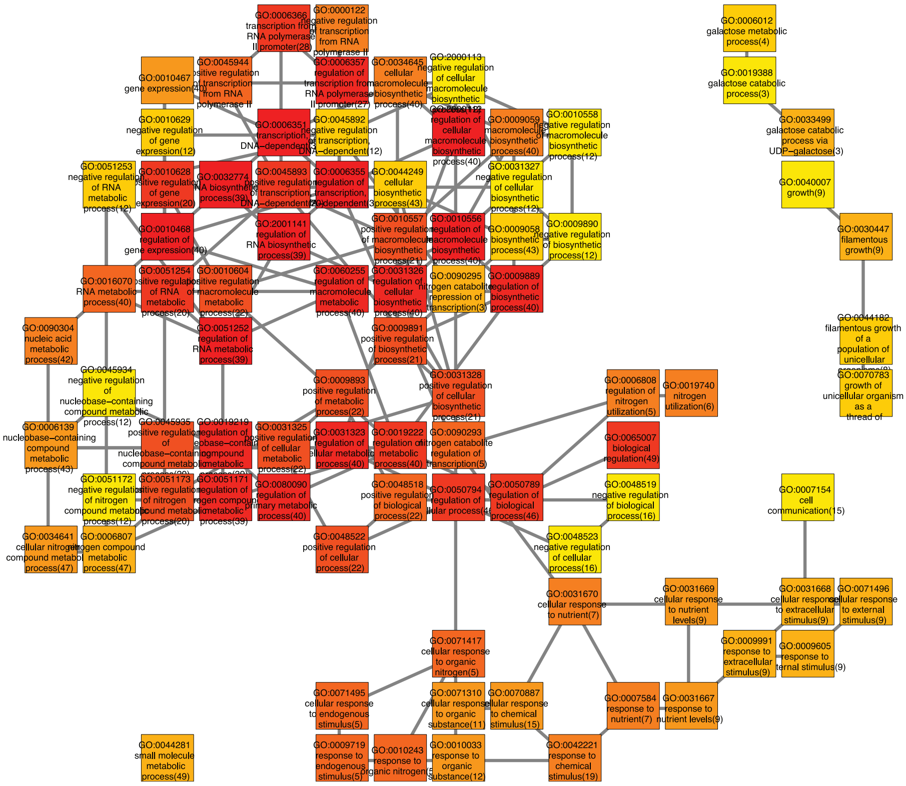

Figure 1: Gene Ontology Biological Process enrichment analysis of the *S. cerevisiae* A) GRN-PPN interface (785 genes, 82 terms $p_{fdr} \leq 0.01$) and B) GRN-TRN interface (64 genes, 86 terms $p_{fdr} \leq 0.01$).

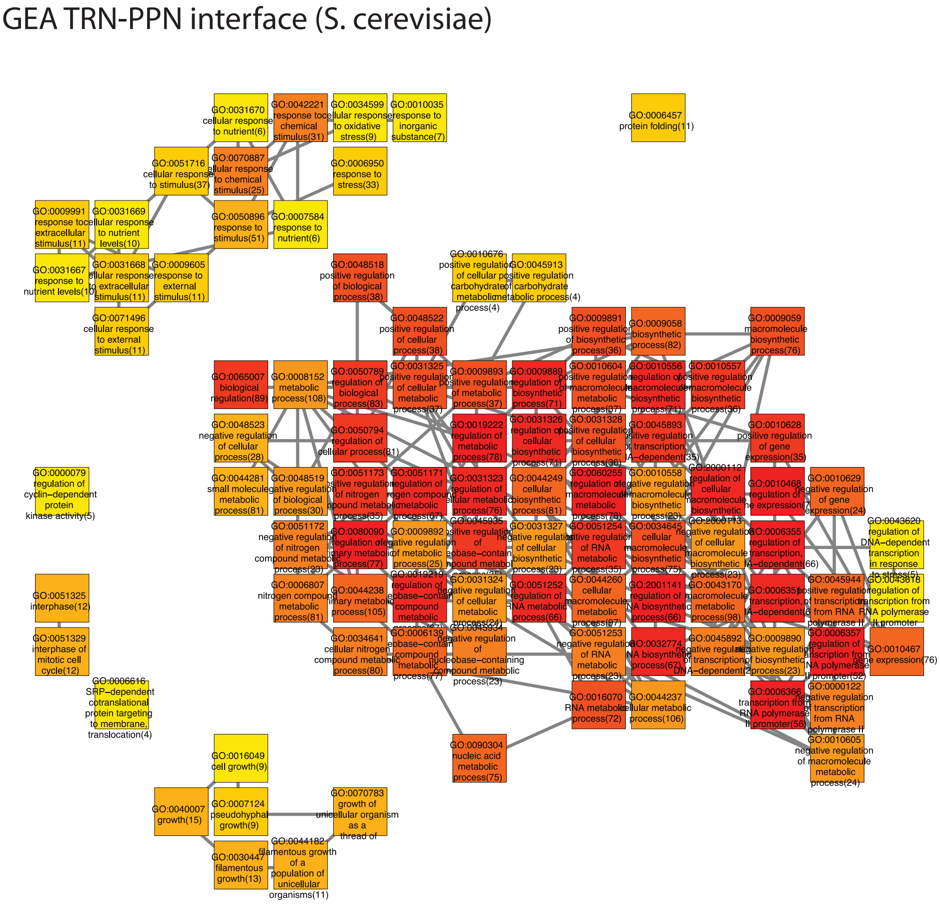

Figure 2: Gene Ontology Biological Process enrichment analysis of the *S. cerevisiae* TRN-PPN interface (115 genes, 96 terms $p_{fdr} \leq 0.01$).

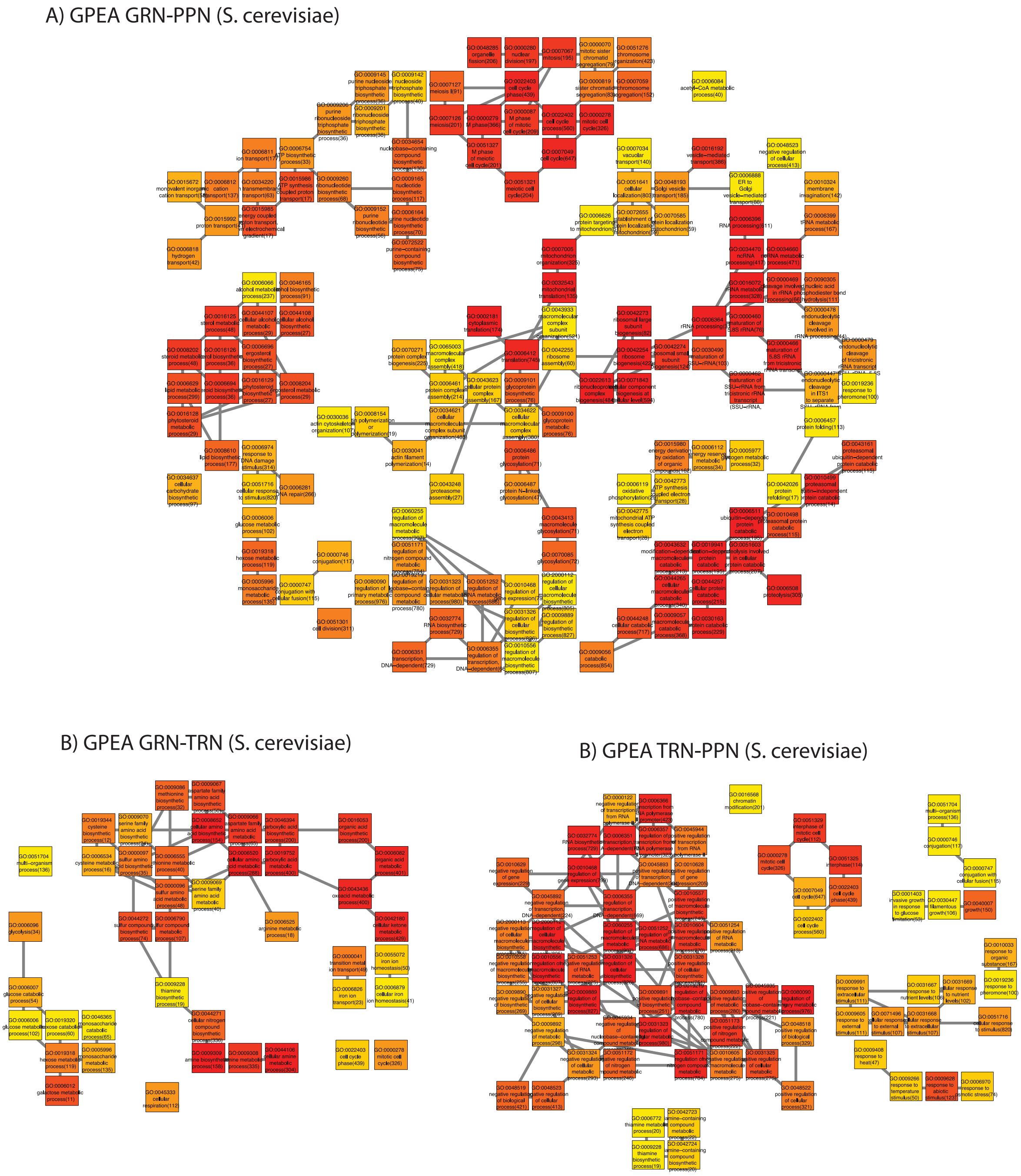

Figure 3: Gene Pair enrichment analysis of the *S. cerevisiae* GRN, PPN and TRN network. Shown are the shared significant Gene Ontology terms for A) GRN-PPN (top 150 terms from 249 $p_{bonf} \leq 10e-4$) B) GRN-TRN 42 terms ($p_{bonf} \leq 10e-4$) and C) TRN-PPN 75 terms ($p_{bonf} \leq 10e-4$). In brackets after the term name shown the number of edges in the respective term for network 1 and network 2.

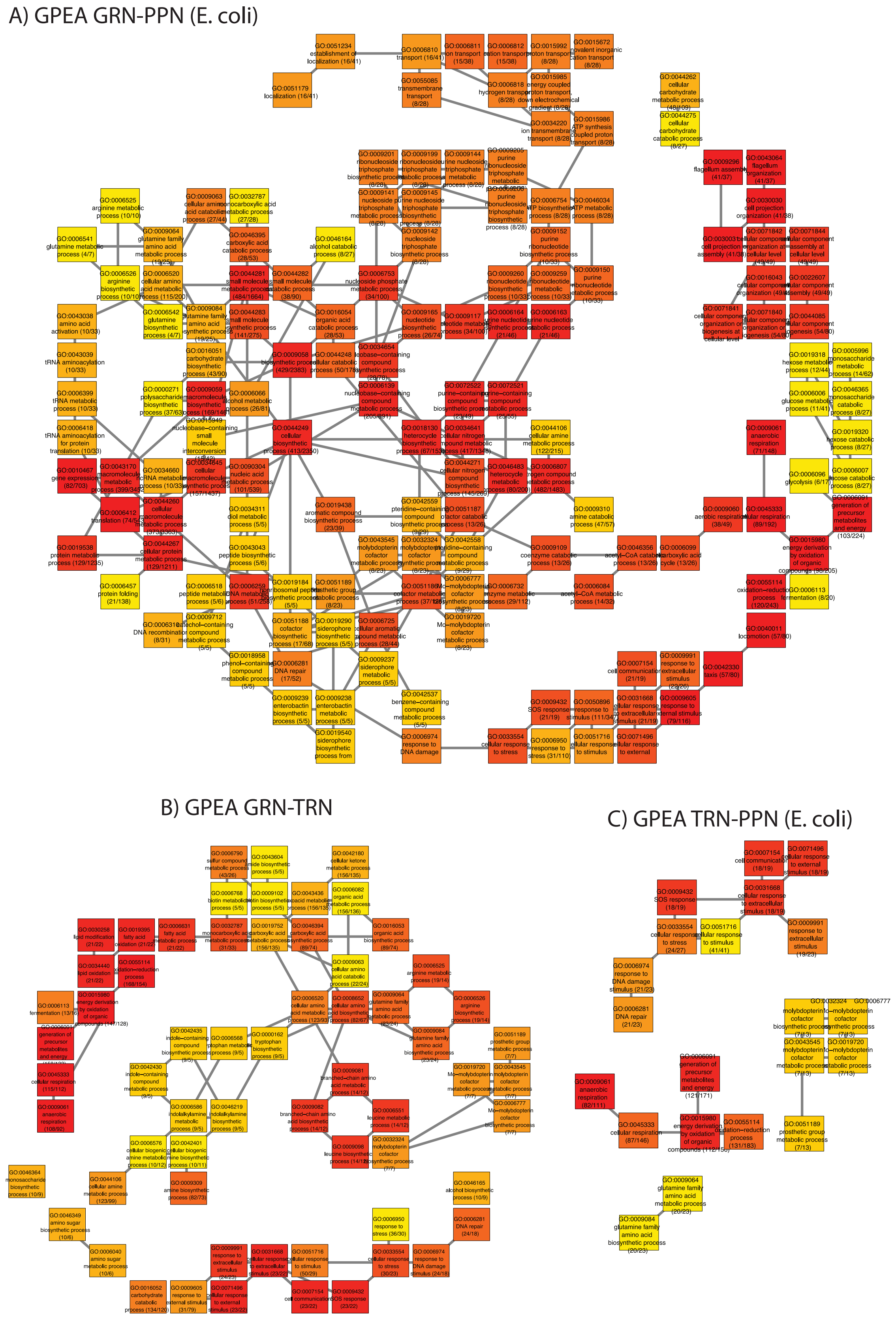

Figure 4: Gene Pair enrichment analysis of the *E. coli* GRN, PPN and TRN network. Shown are the shared significant Gene Ontology terms for A) GRN-PPN (151 terms $p_{bonf} \leq 10e-4$) B) GRN-TRN 63 terms ($p_{bonf} \leq 10e-4$) and C) TRN-PPN 21 terms ($p_{bonf} \leq 10e-4$). In brackets after the term name shown the number of edges in the respective term for network 1 and network 2.

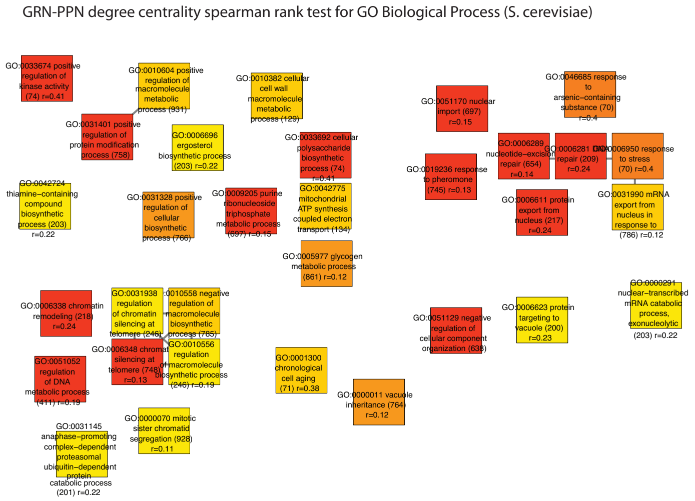

Figure 5: GRN-PPN degree centrality Spearman's rank test for GO Biological Process (*S. cerevisiae*). In brackets shown the number of genes shared between both networks of the corresponding GO term sub-networks and the centrality Spearman's correlation coefficient ρ. Color gradient (red to yellow) denote the rank of the 32 shown terms with $p_{fdr} \leq 0.05$.
